# Endogenous Viral Elements in Animal Genomes

**DOI:** 10.1371/journal.pgen.1001191

**Published:** 2010-11-18

**Authors:** Aris Katzourakis, Robert J. Gifford

**Affiliations:** 1 Department of Zoology, University of Oxford, Oxford, United Kingdom; 2 Aaron Diamond AIDS Research Center, New York, New York, United States of America; Fred Hutchinson Cancer Research Center, United States of America

## Abstract

Integration into the nuclear genome of germ line cells can lead to vertical inheritance of retroviral genes as host alleles. For other viruses, germ line integration has only rarely been documented. Nonetheless, we identified endogenous viral elements (EVEs) derived from ten non-retroviral families by systematic *in silico* screening of animal genomes, including the first endogenous representatives of double-stranded RNA, reverse-transcribing DNA, and segmented RNA viruses, and the first endogenous DNA viruses in mammalian genomes. Phylogenetic and genomic analysis of EVEs across multiple host species revealed novel information about the origin and evolution of diverse virus groups. Furthermore, several of the elements identified here encode intact open reading frames or are expressed as mRNA. For one element in the primate lineage, we provide statistically robust evidence for exaptation. Our findings establish that genetic material derived from all known viral genome types and replication strategies can enter the animal germ line, greatly broadening the scope of paleovirological studies and indicating a more significant evolutionary role for gene flow from virus to animal genomes than has previously been recognized.

## Author Summary

The presence of retrovirus sequences in animal genomes has been recognized since the 1970s, but is readily explained by the fact that these viruses integrate into chromosomal DNA as part of their normal replication cycle. Unexpectedly, however, we identified a large and diverse population of sequences in animal genomes that are derived from non-retroviral viruses. Analysis of these sequences—which represent all known virus genome types and replication strategies—reveals new information about the evolutionary history of viruses, in many cases providing the first and only direct evidence for their ancient origins. Additionally, we provide evidence that the functionality of one of these sequences has been maintained in the host genome over many millions of years, raising the possibility that captured viral sequences may have played a larger than expected role in host evolution.

## Introduction

Viral infection of germ line cells (i.e. gametes, or cells of the early embryo) can lead to viral genes or genomes becoming integrated into chromosomes and inherited as host alleles [1], [2]. These insertions, which we refer to here as *endogenous viral elements* (EVEs), are usually eliminated from the host gene pool within a small number of generations. However, they can also increase in frequency, and some eventually reach fixation [3]–[11].

**Figure 1:**
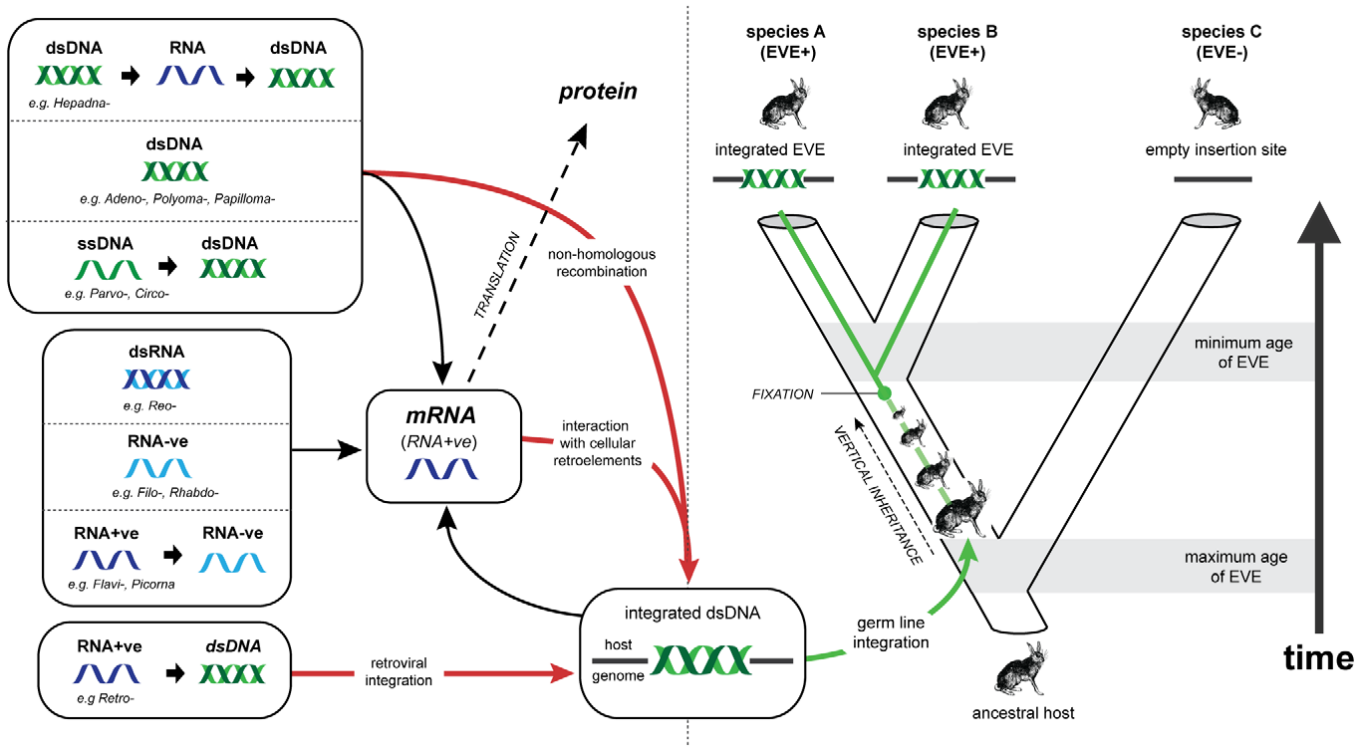
Viral replication strategies, endogenous viral elements, and the genomic fossil record. Animal viruses exhibit a range of genome types and replication strategies. While all viruses must produce mRNA in order to express proteins, steps between entry into the cell and the expression of mRNA vary greatly. Examples of the known animal virus replication strategies are shown to the left of the figure, with the representative families listed for each case. Arrows indicate steps in replication. Red lines indicate pathways that lead to viral genetic material becoming integrated into the nuclear genome of the host cell. Retroviruses are unique amongst animal viruses in that integration occurs as an obligate step in replication. For all other animal viruses integration occurs anomalously, through interaction with cellular retroelements such as LINEs, or via non-homologous recombination with genomic DNA. If integration occurs in a germ line cell that goes on to develop into a viable host organism, an EVE is formed. Green lines show the evolution of an EVE in its host lineage. In the example given, the EVE reaches genetic fixation at the point indicated, and is inherited by all descendant hosts thereafter. Assuming that insertion occurs randomly, the presence of related EVEs at the same locus in both descendant species A and B indicates that insertion occurred prior to their divergence, allowing a minimum age for the insertion to be inferred from the estimated timescale of their evolution. Conversely, the presence of an empty insertion site in species C provides a maximum age for the insertion. Abbreviations: dsDNA (double stranded DNA); ssDNA (single stranded DNA, dsRNA (double stranded RNA); RNA-ve (negative sense, single stranded RNA); RNA-ve (negative sense, single stranded RNA); RNA+ve (positive sense, single stranded RNA).

In animal genomes, the majority of EVEs are derived from reverse transcribing RNA (rtRNA) viruses (i.e. retroviruses) [5], [12], [13]. Retroviruses are the only animal viruses that integrate into the genome of the host cell as an obligate step in their replication strategy, and are thus predisposed to enter the host germ line (Figure 1). EVEs derived from viruses that use other genome replication strategies also occur, but are much less common [6], [7], [9], [11], [14], [15]. Genomic integration of non-retroviral viruses may be mediated by non-homologous recombination with chromosomal DNA [16]–[18] or by interactions with retroelements in the host cell [11], [19]–[22] (Figure 1).

EVEs reveal complex evolutionary relationships between viruses and their hosts. For example, endogenous retroviruses have shaped vertebrate genome evolution, not only by acting as genetic parasites [23], [24], but also by introducing useful genetic novelty. Indeed, the role of *exapted* retroviral genes (i.e. integrated retroviral genes that have adapted to serve a function in the host genome) in mammalian reproduction [25], [26] identifies EVEs as a key factor in the evolution of placental mammals from egg-laying ancestors. Similarly, in parasitoid wasps, genes derived from ancestral nudiviruses have been exapted to facilitate a parasitic lifestyle [9]. These remarkable examples demonstrate an important role for gene flow from viruses to hosts in animal evolution.

EVEs also constitute an invaluable resource for reconstructing the long-term history of virus and host evolution [27], [28]. Viruses exhibit the potential for extremely high rates of nucleotide substitution, host switching, and lineage extinction, and this sets limitations on what can be reliably inferred from observations of contemporary isolates [29], [30]. EVE sequences effectively represent the ‘molecular fossils’ of ancient viral genomes, preserving information about ancient virus and host interactions that would otherwise be difficult, if not impossible, to infer. For example, EVEs are subject to host rates of evolution and can thus be dated relatively reliably with molecular clock-based approaches, in which genetic divergence correlates linearly with time [31]. In contrast, structural constraints in exogenous viruses may lead to the decoupling of short and long-term rates of viral evolution, rendering molecular clock assumptions unusable over longer timescales [30], [32]–[34]. Furthermore, the identification of orthologous EVE insertions allows the incorporation of independent age estimates based on host species divergences (see Figure 1) [35].

Despite the large quantity of published genome sequence data, the diversity of non-retroviral viruses in animal genomes has not been systematically explored. In this report, we use an *in silico* approach to screen the genomes of mammals, birds and insect vector species for endogenous sequences derived from non-retroviral mammalian viruses. We identify sequences derived from a very broad range of viruses, revealing an extensive history of non-retroviral genome invasion ranging back to at least the late Mesozoic Era (∼93 million years ago). We demonstrate that these sequences can be highly informative; (i) revealing novel virus diversity; (ii) providing a timescale for virus evolution; (iii) indicating the likely host range of virus groups, and; (iv) identifying rare instances of horizontal transmission. Furthermore, using a novel approach, we show that at least some of the EVE sequences identified here are likely to have been exapted during their evolution. The implications of these findings are discussed.

## Results

### Genome screening

An algorithm for *in silico* screening of genomes for endogenous non-retroviral insertions was developed. We selected all non-retroviral virus genera that infect mammals, and constructed a library of representative peptide sequences (restricted to viruses with typical genome sizes of <100 Kilobases (Kb)) (Table S2). The tBLASTn program was used to screen low coverage and complete genome assemblies for sequences exhibiting similarity to viral peptides in this library. We screened the genomes of likely reservoirs (birds, n = 2) and vector species (mosquitoes, n = 3; ticks, n = 1) as well as all available mammal genomes (n = 44) (Table S1). Sequences that matched viral peptides with e-values <0.001 were extracted (along with flanking sequences) and putative protein sequences were inferred through a combination of automated and manual alignment. These sequences were assigned to taxonomic groups (family, genus) based on the most closely related exogenous viral sequences in searches of PFAM and Genbank databases (Tables S3, S4, S5, S6, S7). For EVEs that were found to encode uninterrupted open reading frames (ORFs), putative protein sequences were used with the tBLASTn program to search expressed sequence tag (EST) databases for the corresponding mRNA. For all EVEs disclosing similarity to contemporary virus isolates, putative EVE protein sequences were aligned with representative viral protein sequences, and maximum likelihood phylogenies were constructed.

### EVEs related to viruses with RNA genomes

We identified numerous, highly significant matches (i.e. e-values <1×10^−9^) to RNA viruses in the genomes of mammals and insect vectors (Table 1, Tables S3, S4, S5). EVEs related to a total of seven families were identified including double stranded RNA (dsRNA) viruses (*Reoviridae*) and positive sense RNA (RNA+ve) viruses (*Flaviviridae*), as well as both segmented (*Orthomyxoviridae*, *Bunyaviridae*) and non-segmented (*Borna*-, *Filo*- and *Rhabdoviridae*) families of negative sense RNA (RNA-ve) viruses. Consistent with an integration process involving viral mRNA (rather than genomic RNA), all EVEs derived from RNA viruses had genetic structures that spanned a single viral transcript (or fragments derived from single transcripts). EVEs derived from different genes never occurred as contiguous sequences, and consequently we could not determine whether EVEs derived from distinct genes of a given virus family originated from the same or distinct virus lineages/infections.

**Table 1:** Distribution and diversity of EVEs identified by *in silico* screening.

Family (genus)	Replication	Exogenous host range	EVE host classes	EVE loci *^a^*
**DNA viruses**				
*Parvoviridae* *	Nuclear	Mammals, birds	Mammals	99 (11)
**Dependovirus**	-	Mammals, birds	-	57 (5)
Parvovirus	-	Mammals	-	41 (6)
Amdovirus	-	Mammals	-	1
*Circoviridae* *	Nuclear	Mammals, birds	Mammals	5
Hepadnaviridae*	Nuclear	Mammals, birds	Birds	8
**RNA viruses**				
Bornaviridae*	Nuclear	Mammals, birds	Mammals	67
*Filoviridae*	Cytoplasmic	Mammals	Mammals	25
*Bunyaviridae*	Cytoplasmic	Vertebrates, insects	Insects	40
Nairovirus	-	Vertebrates, insects	-	31
Phlebovirus	-	Mammals, insects	-	9
Rhabdoviridae*	Cytoplasmic	Mammals, birds, insects	Insects	143
*Orthomyxoviridae*	Nuclear	Mammals, birds, insects	Insects	1
*Reoviridae*	Cytoplasmic	Mammals, birds, insects	Insects	1
Flaviviridae*	Cytoplasmic	Mammals, birds, insects	Insects	5
*Unclassifiable*	N/A	N/A	Mammals	2

**Footnote:** Groups that have been reported as mediating their own integration are highlighted in bold. Asterisks denote families with representatives known to be capable of establishing persistent/latent infection. EVE insertions were considered to represent complete viral genomes or genes if more than 90% of the species-specific gene/genome was identified. Orthologous insertions in distinct species were not counted as distinct elements. Sequences that were identified by BLAST, but where the region used in phylogenetic trees did not match any viral sequences in reciprocal BLAST searches, were defined as unclassifiable. *^a^* Numbers in parentheses indicate complete genomes.

In mammals, matches to RNA virus proteins that spanned complete genes were typically flanked by target site duplications (TSDs) and 3′ poly-A tails, consistent with LINE-mediated retrotransposition of viral mRNAs [36]. In insects, similar features were not apparent for any EVE insertion, even when the boundaries of host and viral sequences were clearly identifiable (Figure S1). Notably, putative 3′ poly-A tails could be identified in the expected position for some mammal genome sequences that matched only weakly to RNA virus peptides, suggesting the presence of EVEs at the limit of detection to our search strategy.

#### EVEs related to RNA-ve viruses

**Figure 2:**
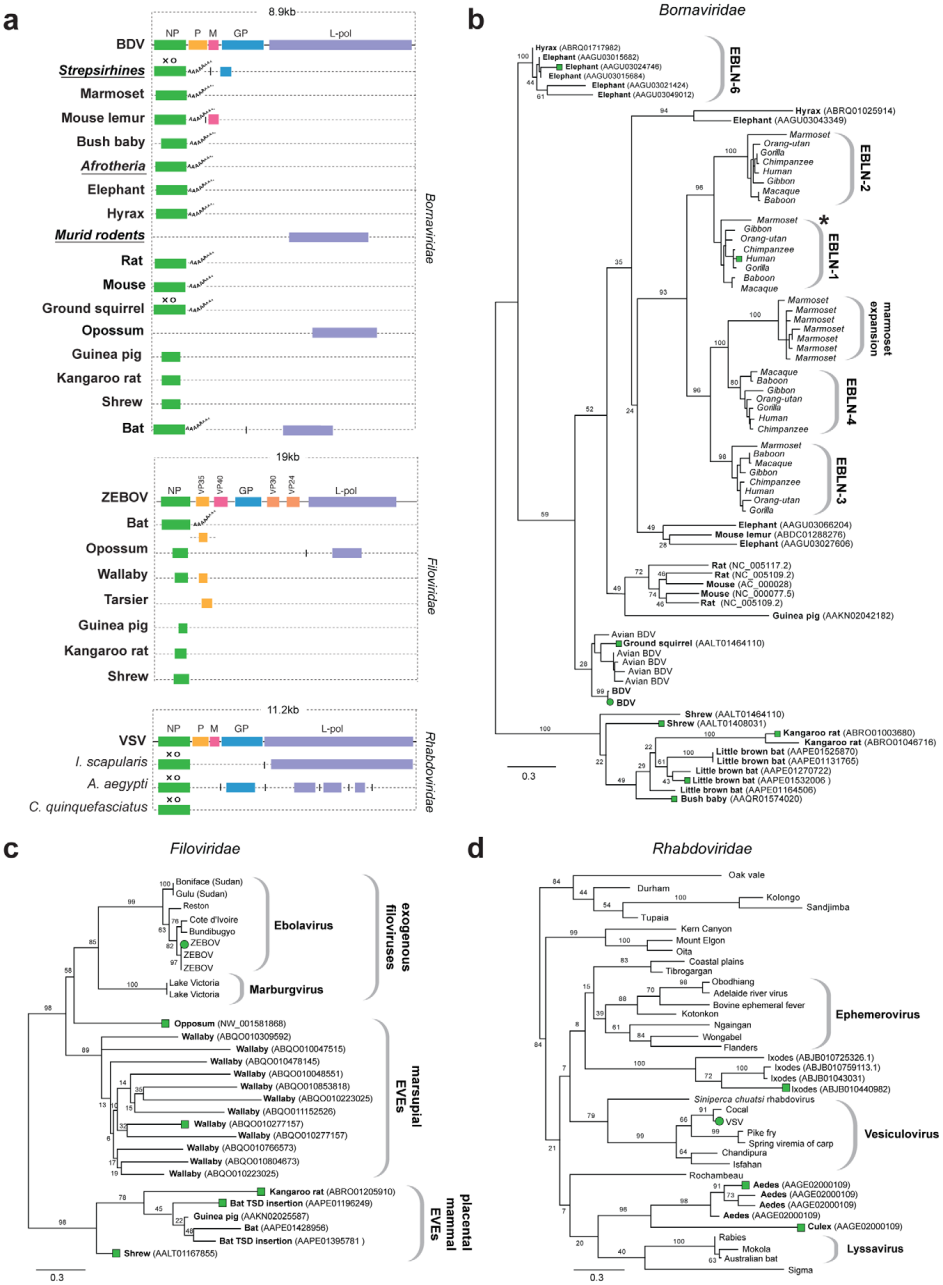
Genetic structures and phylogenetic relationships of Mononegavirus EVEs. (a) Summary genetic structures of Mononegavirus EVE sets (*Borna*-, *Rhabdo*- and *Filoviridae*) shown relative to genus type species. The most intact elements are shown for each host taxon. Vertical lines between EVEs in the same host species that are derived from distinct genes indicate that the EVEs are not contiguous in the host genome. Abbreviations for viral type species (bold), host species (italics), and host taxa (bold, italic, underline) are indicated to the left of each EVE. Taxonomic groups are shown for EVE insertions identified as orthologs. Poly-A tails are shown for EVEs that had these features. Intact ORFs (circles) and expressed sequences (crosses) are indicated. Phylogenetic relationships of (b) bornavirus, (c) rhabdovirus and (d) filovirus EVEs and representative exogenous viruses. Taxa that are shown as genetic structures in (a) are indicated by colored squares. Support for the ML phylogenetic trees was evaluated using 1,000 nonparametric bootstrap replicates, and all three trees are midpoint rooted for display purposes. Abbreviations: BDV = Borna disease virus; ZEBOV = Zaire ebola virus; VSV = vesicular stomatitis virus, L-pol = L-polymerase.

Numerous EVE sequences disclosing similarity to proteins derived from non-segmented RNA-ve virus families in the order *Mononegavirales* (*Borna*, *Filo* and *Rhabdoviridae*) were identified. Matches to genes encoding the relatively conserved nucleoprotein (NP) and L-polymerase proteins predominated, but matches to more rapidly evolving glycoproteins were also identified (Figure 2a). A subset of RNA-virus related EVEs identified in the wallaby genome (Table 1) exhibited significant similarity to a mononegaviral RNA polymerase but were only distantly related to any known group and were not analyzed further.

Sequences disclosing similarity to bornavirus proteins were identified in 16 mammalian genomes. In addition to numerous EVEs derived from bornavirus NP genes (some of which have previously been reported as endogenous Borna-like N (EBLN) elements [11]), we identified EVEs derived from bornavirus L-polymerase, matrix (M) and glycoprotein (GP) genes (Figure 2a). Several sets of orthologous insertions were identified; (i) four distinct NP insertions in strepsirrhine primate genomes (previously been reported as EBLN 1–4 [11]); (ii) GP in strepsirrhine primates; (ii) L-polymerase in rats and mice; (iii) NP in three Afrotherian species (African elephant, cape hyrax, lesser hedgehog tenrec (*Echinops telfairi*)). Bornavirus nucleoprotein trees revealed an expansion of EBLNs in the New World branch of strepsirrhine primates, represented by the common marmoset (*Callithrix jacchus*) (Figure 2b).

Sequences matching filovirus NP genes were identified in the little brown bat (*Myotis lucifugus*), Tammar wallaby (*Macropus eugenii*), and gray short-tailed opossum (*Monodelphis domestica*) genomes. The majority of these matches comprised fragments of genes, although two full-length NP gene EVEs (displaying poly-A tails and TSDs) were identified in bats (Figure 2a). More divergent fragments of the NP gene were identified in the kangaroo rat (*Dipodymys ordii*), guinea pig (*Cavia porcellus*) and common shrew (*Sorex araneus*) genomes. Additionally, fragments of the L-polymerase and VP35 gene were identified in the genomes of the opossum and Philippine tarsier (*Tarsius syrichta*) respectively. In phylogenies, EVEs derived from filovirus NP genes grouped into two well-supported clades (Figure 2c), the largest of which included exogenous filoviruses and EVEs derived from marsupials (wallaby and opossum). EVEs in the smaller clade were more distantly related to extant filoviruses, and were derived from the little brown bat, guinea pig, shrew and kangaroo rat genomes. An opossum EVE derived from L-polymerase grouped relatively closely with Marburgviruses. Conflicting phylogenetic trees for oppossum L-polymerase and NP-derived EVEs strongly indicated they are derived from distinct ancestral viruses.

EVEs related to rhabdoviruses were identified in the black-legged tick genome (*Ixodes scapularis*), and in the genomes of both *Aedes* and *Culex* mosquitoes. Among these were insertions that encoded intact NP and GP ORFs (Figure 2a). Phylogenies constructed using NP tentatively grouped rhabdovirus EVEs derived from mosquitoes in a clade with lyssaviruses and *Drosophila* sigma virus (Figure 2d). However phylogenetic support for this clade was very weak, with only 20% bootstrap support for the monophyly of the clade, although support for the grouping of EVEs from *Aedes* and *Culex* was high (96%). A robust clade (100% bootstrap support) placed four *Ixodes* EVEs into a single group, suggesting they are likely derived from the same exogenous virus lineage, but their placement relative to other Rhabdoviruses was ambiguous, as they formed a clade with a number of distinct Rhabdoviruses with minimal bootstrap support (8%). Phylogenies constructed using L-polymerase sequences weakly grouped *Ixodes* and *Aedes* insertions with Lyssaviruses and Moussa virus, but not *Drosophila* sigma virus. Weak support for basal relationships was obtained with both trees, making it difficult to confidently place thenovel EVEs with respect to the known rhabdovirus diversity.

**Figure 3:**
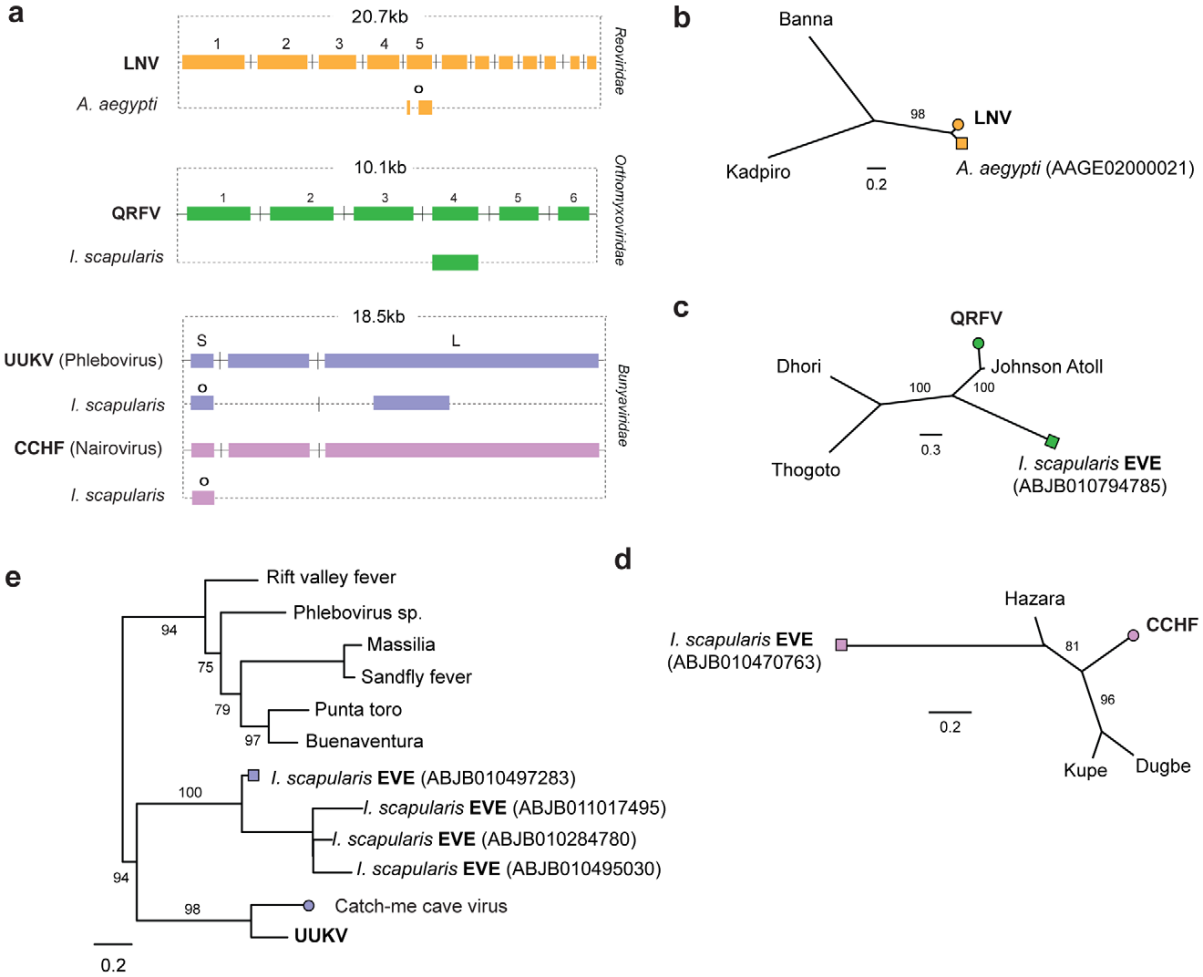
Genetic structures and phylogenetic relationships of EVEs related to segmented RNA viruses. Summary genetic structures of EVEs derived from segmented RNA viruses (a) *Reoviridae* (Seadornavirus genus), (b) *Orthomyxoviridae* (Quarjavirus genus), (c) *Bunyaviridae* (Nairovirus and Phlebovirus genera)) shown relative to the genus type species. The most intact elements are shown for each host taxon. Intact ORFs (circles) and expressed sequences (crosses) are indicated. Maximum likelihood phylogenies of EVEs and exogenous viruses are shown; (d) *Reoviridae* (segment 5) (e) *Orthomyxoviridae* (GP), (f) Nairovirus (NP) (g) Phlebovirus (NP) Colored boxes indicate taxa that are shown as genetic structures in panels a-c. Support for trees was evaluated using 1,000 nonparametric bootstrap replicates. Abbreviations: LNV = Liaoning virus; CCHF = Crimean-Congo hemorrhagic fever virus; UUKV = Uukuniemi virus; QRFV = Quaranfil virus.

Matches to RNA-ve viruses with segmented genomes were identified in the genomes of insect vectors (Figure 3a). In the *I. scapularis* genome, we identified EVEs related to viruses isolated from ticks and birds basal to the proposed genus Quarjavirus (including Quaranfil and Johnston Atoll viruses) [37] in the family *Orthomyxoviridae* (Figure 3c), EVEs distantly related to the *Bunyaviridae* (Phlebovirus and Nairovirus genera) were identified in the *I. scapularis* genome. Nairovirus-derived EVEs were distantly related to Hazara virus (Figure 3d), indicating they represent a distinct lineage within this tick-vectored genus. Phlebovirus EVEs formed a robustly supported cluster in phylogenies with exogenous viruses (Figure 3e), closest to Uukuniemi and Catch-me cave viruses (vectored by ticks and mosquitoes respectively), suggesting they are derived from the same exogenous lineage.

#### EVEs related to dsRNA viruses

In the *A. aegyptii* genome we identified an EVE that was very closely related (∼98% nucleotide sequence identity) to segment 5 of the Liaoning virus genome (Figure S1, Figure 3a and 3b). Liaoning is a dsRNA virus (family *Reoviridae*, genus Seadornavirus) that was recently isolated from *Aedes dorsalis* mosquitoes [38]. The Liaoning EVE in *A. aegyptii* had a large inframe deletion, but encoded an otherwise intact ORF. This is the first EVE derived from a dsRNA virus to be described. As with other RNA virus EVEs in insect genomes, the mechanism of genomic integration was unclear. The intact ORFs and high level of identity to a circulating virus raise the possibility this EVE formed recently and is not fixed in the host population.

#### EVEs related to RNA+ve viruses

**Figure 4:**
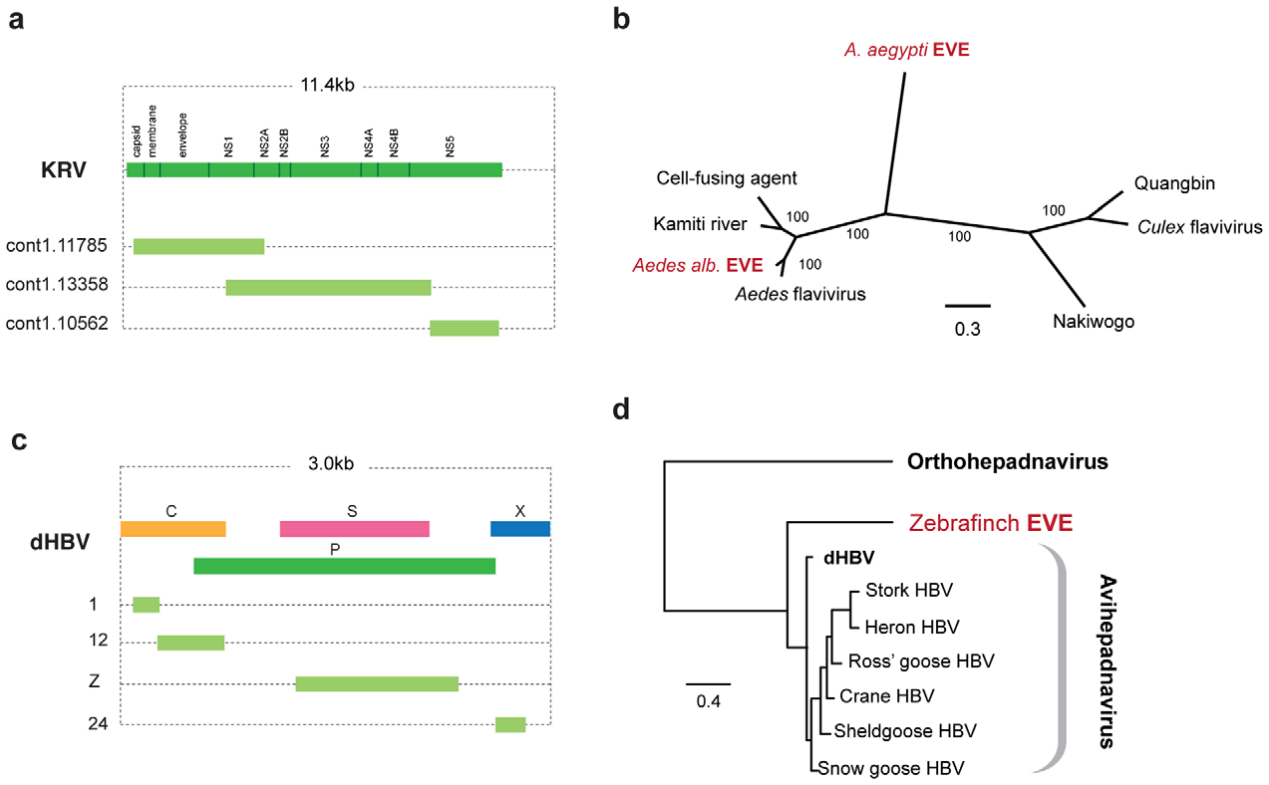
Genetic structures and phylogenetic relationships of EVEs related to flaviviruses and hepadnaviruses. (a) Genetic structures of non-overlapping flavivirus EVEs in the *Aedes aegyptii* genome. (b) Phylogenetic relationship of consensus flavivirus EVE sequences (spanning most of the region shown in (a) with exogenous and endogenous flaviruses. (c) Genetic structures of non-overlapping rtDNA (hepadnavirus) EVEs shown relative to the genus type species. Numbers to the left indicate the *T. guttata* chromosome on which the EVE is present. (d) Phylogenetic relationships of consensus zebrafinch EVEs and representative exogenous viruses. Avihepadnavirus genus is rooted on Woodchuck HBV (Orthohepadnavirus). All ML phylogenetic trees were inferred from amino acid alignments using the best-fitting model of evolution. Support for trees was evaluated using 1,000 nonparametric bootstrap replicates. Abbreviations: KRV = Kamiti River virus, HBV = hepatitis B virus, dHBV = duck hepatitis B virus.

The genome of the *Aedes* mosquito contains several sequences exhibiting similarity to the viruses of the RNA+ve family *Flaviviridae*. Endogenous flaviviruses have previously been reported in the genomes of *A. aegyptii* and *A. albopictus* mosquitoes [6], [39], but complete putative genomic structures have not been determined. In particular, a large fragment spanning the flaviviral NS1, NS2A, NS2B, NS3 and NS4A genes has been described in *A. albopictus*, and a range of smaller fragments at the 3′ end of the flaviviral genome, mostly of the NS5 gene, have been described in both *albopictus* and *aegyptii* species. We have identified fragments that together span almost the entire flavivirus genome in *A. aegyptii* (based on alignment to Kamiti river virus (Figure 4a)), including a single fragment that spans the equivalent region from the *albopictus* genome. Phylogenetic trees that included both *aegyptii* and *albopictus* EVEs showed that they are distinct viruses, separated by known exogenous isolates. Thus, the EVEs in these two mosquito species appear to be derived from at least two distinct flavivirus lineages, with the *aegyptii* virus being most divergent from previously characterized isolates. The *albopictus* sequence grouped in a clade that included both Kamiti river virus and cell fusing agent, as previously described by Crochu *et al*. [6].

### EVEs related to viruses with DNA genomes

We identified highly significant matches to three families of viruses with DNA genomes in the genomes of mammals and birds (Table 1, Tables S6 and S7). These included matches to two single stranded DNA (ssDNA) virus families (*Parvoviridae* and *Circoviridae* - the first ssDNA virus EVEs to be described in mammals - and one family of reverse transcribing DNA (rtDNA) viruses (*Hepadnaviridae*) - the first rtDNA EVEs to be described. A single match to a double stranded DNA (dsDNA) virus family (*Adenoviridae*) was identified in the kangaroo rat genome, but this sequence was unambiguously viral across its entire length (∼17 Kb), encoding thirteen completely intact viral ORFs (Figure S2), and is thus likely to have derived from free virus and not an EVE.

**Figure 5:**
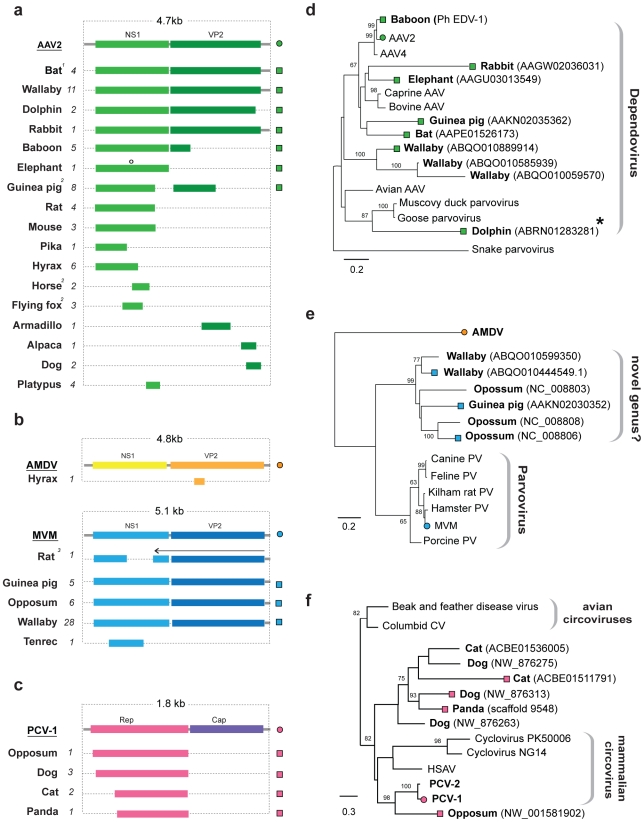
Genetic structures and phylogenetic relationships of EVEs related to ssDNA viruses. (a) Summary genetic structures of ssDNA EVE sets shown relative to the genus type species. The most intact elements are shown for each host taxon. EVE hosts (bold) and abbreviations for viral type species (bold, underline), and the total number of matches identified (italic) are indicated to the left of each EVE structure. Bars behind ORFs indicate non-coding viral DNA. Intact ORFs (circles) and expressed sequences (crosses) are indicated. ^1^ The *M. lucifugus* element is a composite of two genomic contigs; ^2^ Structures represent a composite of semi-overlapping fragments; ^3^ Element has undergone genomic rearrangements, with the arrow indicating the direction of the rearranged fragment. (b) Phylogenetic relationships of dependovirus EVEs and representative exogenous viruses, based on NS1 gene and rooted on snake parvovirus. A dolphin EVE (indicated by an asterisk) groups robustly with avian rather than mammalian isolates. (d) Phylogenetic relationships of parvovirus EVEs and representative exogenous viruses, based on NS1 gene and rooted on Aleutian mink disease virus. Support for both ML phylogenetic trees was evaluated using 1,000 nonparametric bootstrap replicates. EVEs potentially comprising a new genus are indicated. (d) Phylogenetic relationships of circovirus EVEs and representative exogenous viruses, based on the Rep gene and rooted on avian circoviruses, with support for the ML phylogenetic tree evaluated using 1,000 nonparametric bootstrap replicates. Taxa that are shown as genetic structures in (a) are indicated by colored squares (EVEs) and circles (exogenous viruses) (a). Abbreviations: AAV = adeno-associated virus; MMV = minute virus of mice, AMDV = Aleutian mink disease virus, PV = parvovirus, CV = circovirus, PCV = porcine circovirus; HSAV =  Human stool-associated circular virus.

A subset of parvovirus-related EVEs represented complete or nearly complete viral genomes (Figure 5a). For one insertion in the *M. lucifugus* genome, we identified putative 5′ and 3′ terminal non-coding regions encoding characteristic inverted terminal repeats (Figure S3). In general, however, DNA virus EVEs occurred as genomic fragments, with no particular region of the viral genome being obviously favored, with the exception of the circoviruses, for which only the Rep gene was found.

#### EVEs related to rtDNA viruses

We identified sequences disclosing significant similarity to Hepadnaviruses in the genomes of the black-legged tick (*I. scapularis*), and the zebrafinch (*Taeniopygia guttata*). In the zebrafinch genome, a total of 7 loci were identified on 7 distinct chromosomes (Figure 4c). Sequences at each locus generally corresponded to distinct, non-overlapping regions of viral genome, suggesting that host genome arrangements had fragmented a more complete insertion, spanning most if not all of the viral genome. A consensus constructed using all 7 zebrafinch hepadnavirus EVE insertions, and representing ∼80% of the viral genome, grouped with avian hepadnaviruses in maximum likelihood phylogenies (Figure 4d). Although only distantly related to vertebrate hepadnaviruses (and hence not included in phylogenies), matches in the tick genome indicate the existence of an uncharacterized lineage of insect hepadnaviruses.

#### EVEs related to ssDNA viruses

EVEs derived from viruses of the family *Parvoviridae* were identified in a broad range of mammalian genomes (Figure 5a). In total, 58 EVEs in 17 species matched closely to the Dependovirus genus, 41 EVEs in 5 species matched the Parvovirus genus, and a single element in the cape hyrax (*Procavia capensis*) genome matched the Amdovirus genus (Table 1, Table S6). Phylogenies confirmed the majority of these designations, grouping EVEs robustly within the diversity of genera to which they were assigned. However, a group of EVEs identified in the Tammar wallaby, opossum, and guinea pig genomes formed a distinct and well-supported clade, potentially representing a novel genus, intermediate between the Parvovirus and Amdovirus genera (Figure 5e).

The majority of parvovirus EVEs were not intact, and are unlikely to express RNA or protein. However, a dependovirus EVE in the genome of the African elephant (*Loxodonta africana*) encoded an intact NS1 gene (Figure 5a). Additionally, screening of EST databases identified expressed sequences related to an opossum parvovirus EVE in another marsupial species - the brush tailed possum (*Trichosurus vulpecula*).

Adeno-associated virus 2 (AAV-2) integrates at a specific site in human chromosome 19 [40], [41]. Notably, a nearly complete dependovirus EVE identified in the baboon genome, and that grouped closely with AAV-2 in phylogenies (Figure 5e), was inserted at an unambiguously distinct site homologous to human chromosome 21. We identified a dependovirus insertion that was orthologous between rats and mice (Table S6). This insertion comprised fragments of the NS1 gene, with internal breakpoints being bounded by homologous genomic DNA sequences - thus the possibility of two separate, but site-specific integration events could be excluded.

EVEs disclosing significant similarity to the Rep gene of ssDNA family *Circoviridae* were identified in the genomes of the gray short-tailed opossum, and three species of the mammalian order Carnivora; domestic cat (*Felis catus*), dog (*Canis familiaris*) and panda (*Ailuropoda melanoleuca*) (Figure 5c). These sequences grouped with other, recently characterized mammalian circoviruses in phylogenies (Figure 5f). One circovirus insertion was found to be orthologous in all three carnivore species. Host genomic DNA was identified at the 3′ end of the Rep gene, indicating that the downstream Cap gene was absent from these EVEs.

### Paleovirology of EVE insertions

**Figure 6:**
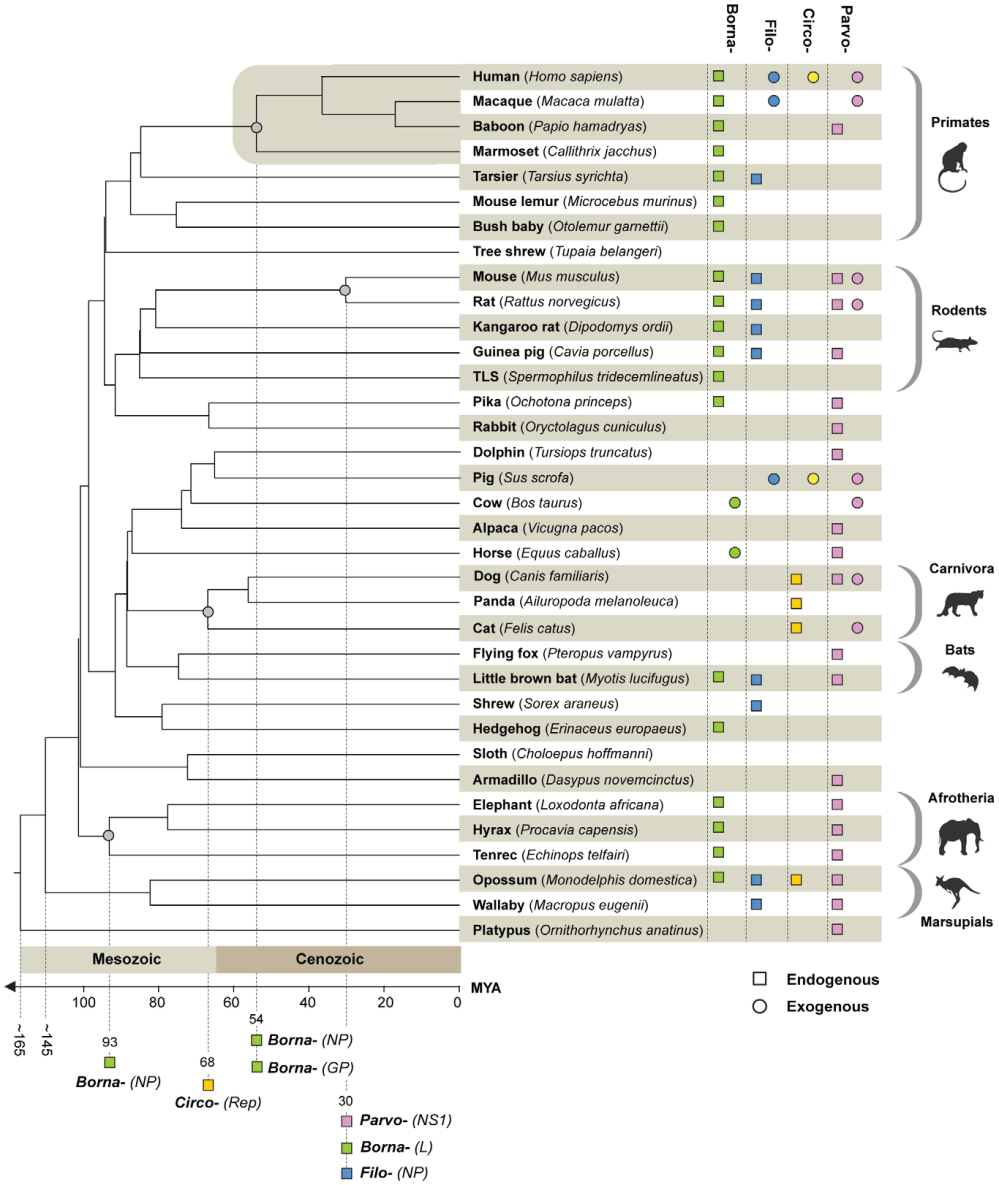
Timescaled phylogenetic tree of mammals screened in this study (after Bininda-Emonds *et al* [**42**]) showing the known distribution of EVEs and of exogenous Borna-, Filo-, Circo-, and Parvoviruses. Grey circles indicate nodes at which orthologous EVE insertions were identified. For all orthologous insertions identified here and elsewhere [11], [22], the virus family and genomic region represented by the ortholog is shown. Abbreviation: TLS = Thirteen-lined ground squirrel.

We identified a number of EVE insertions that were orthologous between species, allowing minimum ages for families to be inferred from host divergence dates (see Figure 2). Using previously estimated mammalian divergence dates [42] we obtained minimum ages for the *Parvo*, *Circo* and *Bornaviridae* of 30, 68 and 93 million years respectively, demonstrating the ancient origins of these families (Figure 6). During completion of this manuscript, orthologous filovirus EVEs were reported in the mouse and rat genomes [22]. These sequences were identified by BLAST searching using EVEs as probes, and were not picked up in our screen, which relied on matches to exogenous viruses. On the basis of the mammalian divergence dates used here [42], these EVEs provide a minimum age of 30 million years for the *Filoviridae* (Figure 6).

The EVEs identified in this study extend the host range of several families (*Parvo-*, *Circo-*, *Hepadna*, *Borna-* and *Filoviridae*) with respect to their known range as exogenous viruses (Figure 6). Dependovirus EVEs are particularly widespread and occur in diverse mammalian hosts, despite their apparent low probability of germ line integration in AAV-derived gene therapy vector *in vivo* models [43]. Filoviruses have only been identified as exogenous infections in bats and primates [44]. However, filoviruses EVEs were identified not only in North American bats (*M. lucifugus*) and Asian primates (tarsier), but also in insectivores, rodents, and in both South American and Australian mammals (Figure 6). In concordance with the recent identification of Ebola Reston in swine [45], this unexpected result indicates that the distribution of filoviruses is likely much broader than has previously been recognized.

Highly discordant host ranges among closely related EVEs (or EVEs and exogenous viruses) can provide information about transmission events. In this regard, we note that a dependovirus EVE in the bottlenose dolphin (*Tursiops truncatus*) genome grouped robustly with avian dependoviruses (rather than mammalian isolates) in NS1 trees (Figure 6d), suggesting cross-class transmission of parvoviruses between birds and mammals may have occurred in the past.

### Evidence for exaptation of EVEs

**Figure 7:**
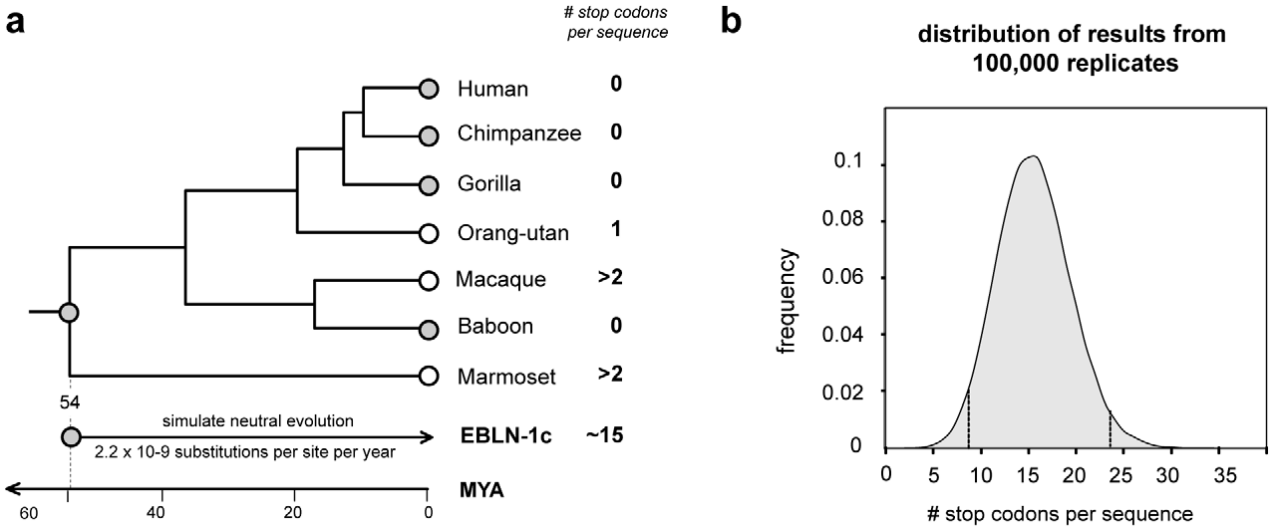
Evolution of EBLN elements in primates. (a) The primate clade marked by an asterisk in the phylogeny shown in Figure 6 is shown in greater detail here, with the number of stop codons in the EBLN-1 locus indicated for seven species. Orthology across these species indicates that EBLN-1 predates the divergence of these species 54 million years ago [42]. Monte Carlo simulations in which a consensus EBLN sequence was allowed to neutrally evolve at the primate neutral rate for this length of time showed that the average number of stop codons expected after this time is fifteen. (b) The distribution of the number of stop codons from 100,000 simulation replicates. Confidence intervals are indicated.

EVEs that are neutral or only slightly deleterious in their hosts may fortuitously drift or hitchhike [46] to fixation, accumulating mutations at the host neutral rate. Alternatively, EVE insertions may confer an advantageous phenotype on the host and spread through the population by selection. In such exapted sequences, selection will act to maintain the functionality of the EVE sequence. Many of the EVEs identified in this study were highly mutated and/or fragmented and these likely represent non-functional, neutrally evolving pseudogenes. However, several EVEs encoded intact ORFs, and some also express RNA (Figure 2a, Figure 3a, Figure 5a). For most of these EVEs, the time since insertion is unknown, and intact ORFs could reflect recent insertion rather than a long-standing history of purifying selection within the host genome. In primates, however, orthology of the bornavirus-derived insert EBLN-1, which is intact in several species, demonstrates an insertion date predating the divergence of strepsirhine primates (∼54 million years ago (MYA)) (Figure 7). Simulations in which a consensus derived from all EBLN-1 sequences was allowed to neutrally evolve over this time period indicated the probability of maintaining an intact ORF in the absence of purifying selection was <0.00001 (100,000 replicates, mean number of stop codons  = 15.57, 95% confidence range 7.9–23.3). This analysis provides more robust support for purifying selection than classical tests based on the ratio of synonymous to non-synonymous mutations (which are weakly significant for EBLN-1 [11]), strongly indicating that EBLN-1 has been exapted in the primate genome, at least during part of its evolutionary history. Curiously, however, EBLN-1 has not retained coding capacity in all primate species. Perhaps selection to maintain it has recently been lost across all primates, and all the inserts may become inactivated in future.

## Discussion

In this report, systematic screening revealed that sequences derived from a broad range of non-retroviral mammalian virus groups occur as endogenous elements in the genomes of mammals, birds and insect vectors. We describe the first EVEs derived from the rtDNA and dsRNA groups, thereby establishing that the complete range of known animal virus replication strategies (see Figure 1) are represented by endogenous elements in animal genomes.

Richer sampling of animal genomes is likely to reveal an even greater diversity of EVEs. While EVEs that are very ancient (i.e. that inserted prior to the divergence of major host lineages) can be identified by selectively screening a small number of host species, identification of more recent insertions will often require richer sampling within orders and genera. Sampling of mammalian species for whole genome sequencing has generally been across, rather than within orders (primates are an exception). Consequently the majority of mammal species sampled in this study diverged more than 50 million years ago (Figure 6). Any mammal species that was not sampled, and diverged more recently, could contain uncharacterized EVEs. Sampling of avian and insect vector genomes has so far been quite limited, and these may also harbor a rich virus fossil history. Furthermore, the vast majority of EVE insertions never reach fixation, and there are likely many unfixed EVEs present within species gene pools at a given time (known examples of unfixed EVEs include Israeli acute paralysis virus (IAPPV) insertions in honey bees (*Apis mellifera*) [15], koala endogenous retrovirus (KoRV) in koala bears [47], and human herpesvirus 6 (HHV-6) and HERV-K HML-2 insertions in humans [18], [48]). Identification of such unfixed EVEs will often require population-level screening.

The *in silico* screening strategy employed here likely underestimates the actual diversity of EVEs for several reasons. Firstly, only low-coverage, incomplete genome data were available for most species. Furthermore, EVEs within the data we screened could have been overlooked because (i) screening was based on similarity searches, and is thus dependent on current (limited) knowledge of viral diversity, and (ii) more ancient EVEs may not be identified due to the divergence in both host and virus lineages subsequent to insertion (this may also result in a bias toward detecting more conserved genes).

Certain groups of (non-retroviral) viruses appear to be better represented in the genomic fossil record than others (e.g. *Parvoviridae*, *Mononegavirales*). This likely reflects a predisposition for germline integration among viruses with particular patterns of replication and infection. Notably, viruses that establish persistent infections and/or replicate within the nucleus are particularly well represented among the EVEs identified in this study. Nevertheless, these characteristics do not appear to be prerequisites for germ line integration (Table 1). Indeed, since retroelements are ubiquitous in animal genomes, and replication of all known viruses requires the expression of RNA, retroelement activity in germ line cells [49] may present a general mechanism for mediating insertion of virus genes into animal germ lines (see Figure 1).

The discovery that a broad range of viruses are represented by EVEs in animal genomes indicates that viral ‘molecular fossils’ can provide the basis for robust, time-scaled, macroevolutionary studies across a range of animal and virus groups. For example, EVE sequences can be combined with phylogenetic data of extant host species to reveal patterns of inter-class virus transmission (Figure 5) [50]. In this study, orthologous EVEs derived from the *Borna*-, *Filo- Circo*-, and *Parvoviridae* provided direct evidence for the ancient origins of these families (Figure 6). These findings also indicate that more recent dates of origin obtained for other virus families using molecular clock-based extrapolations are artifacts [30].

The diversity represented by known virus isolates represents a tiny fraction of the total viral diversity. Indeed, given their likely ancient origins, many virus families may be broadly distributed across mammalian hosts. This was reflected in viral phylogenies containing a mixture of EVEs and exogenous viruses - closely related exogenous relatives could often not be identified, or had only been recently characterized [37], [38], [51] (Figure 2, Figure 3, Figure 5). These findings suggest that EVEs can inform viral surveillance efforts by revealing novel virus diversity and indicating the likely host range of virus groups (particularly if they inserted relatively recently). For example, a strong association between filoviruses and marsupials (Table 1, Figure 2) unexpectedly highlighted this group as a potential filovirus reservoir. The potential presence of EVEs may also be an important consideration in studies where bulk sequencing of environmental samples is used to identify novel virus groups [51]–[53].

EVEs that reach fixation in the host germ line may do so fortuitously, or because they are exapted by the host genome. Monte Carlo simulations provided robust statistical support for a history of purifying selection in the primate EVE EBLN-1, indicating this sequence has been exapted by the primate genome. However, selection on EBLN-1 has clearly relaxed in some primates and may also have relaxed in humans (Figure 7). Such transient co-option may be expected for EVEs that function as restriction factors in their hosts by conferring resistance to infection by exogenous viruses. Several examples of this phenomenon have been described in animals [15], [54], [55], and it is likely one of the most common exaptations of viral genes by host organisms [56], [57]. In these cases, counter-adaptation in a rapidly evolving virus population may eventually render the EVE restriction mechanism non-functional [55], causing selection to relax. Importantly, the rate at which EVEs are exapted as restriction factors in animals could greatly exceed their rate of fixation in animal genomes.

The diverse EVE sequences described in this report demonstrate an extensive history of gene flow from virus to animal genomes. Animal genomes are a living document of virus and host interaction, and genomic studies have an important role to play in advancing understanding of virus and host evolution.

## Materials and Methods

### Genome screening

Chromosome assemblies and whole genome shotgun assemblies of 44 species (Table S1) were screened *in silico* using tBLASTn and a library of representative peptide sequences derived from mammalian virus groups with genomes <100 Kb in total length (selected from the 2009 International Committee on Taxonomy of Viruses (ICTV) master species list (Table S2)). Host genome sequences spanning high-identity (i.e. e-values <0.0001) matches to viral peptides were extracted, and a putative viral ORF was inferred using BlastAlign [58] and manual editing. Putative EVE peptides were then used to screen the Genbank non-redundant (nr) database in a reciprocal tBLASTn search. Matches to retroviruses, viral cloning vectors, and non-specific matches to host loci were filtered and discarded. The remaining sequences were considered viral if they unambiguously matched viral proteins in the Genbank and PFAM databases as shown in Tables S3, S4, S5, S6, S7. Genetic structures for these elements were determined by comparison of the putative EVE peptide sequence to the nucleotide sequence of a viral type species representing the most closely related viral genus recognized by ICTV. Boundaries between viral and genomic regions were identified by analysis of sequences flanking matches to viral peptides, the genomes of the host species, and closely related host species. Sequences that flanked viral insertions were considered genomic if they; (i) were present as empty insertion sites in a related host species; (ii) disclosed highly significant similarity (i.e. e-values <1×10^−9^) to host proteins; or (iii) non-viral and highly repetitive (>50 copies per host genome). Insertions were considered endogenous when >100 bp of genomic flanking sequence could be identified either side of a viral match. Insertions for which >100 bp of unambiguous (i.e. >80% nucleotide identity) flanking sequence was identified in host sister taxa were considered orthologous insertions. PERL scripts were used to automate BLAST searches and sequence extraction. Putative EVE peptide sequences, and alignments of EVEs and exogenous retroviruses, are available online (http://saturn.adarc.org/paleo/).

### Phylogenetic analysis

Putative EVE sequences inferred using BlastAlign were aligned with closely related viruses using MUSCLE and manually edited [59]. Maximum likelihood (ML) phylogenies were estimated using amino acid sequence alignments with RAXML [60], implementing in each case the best fitting substitution model as determined by ProtTest [61]. Support for the ML trees was evaluated with 1000 nonparametric bootstrap replicates. The best fitting models for the datasets were: *Parvoviridae*: dependovirus NS1 gene (JTT+Γ, 332 amino acids across 17 taxa), *Parvoviridae*: parvovirus NS1 gene, (JTT+Γ, 293 amino acids across 13 taxa), *Circoviridae*: Rep gene (Blosum62+Γ+F, 235 amino acids across 14 taxa), *Hepadnaviridae*: polymerase gene (JTT+Γ+F, 661 amino acids across 9 taxa), *Orthomyxoviridae*: GP gene (WAG+Γ+F, 482 amino acids across 5 taxa), *Reoviridae*: VP5 gene (Dayhoff+Γ+F, 171 amino acids across 4 taxa), *Bunyaviridae*: phlebovirus NP gene (LG+Γ, 247 amino acids across 12 taxa), *Bunyaviridae*: nairovirus NP gene (LG+Γ, 446 amino acids across 5 taxa), *Flaviviridae*: mostly NS3 gene (LG+Γ+F, 1846 amino acids across 8 taxa), *Filoviridae*: NP gene (JTT+Γ, 369 amino acids across 29 taxa), *Filoviridae*: L gene (LG+Γ+F, 517 amino acids across 9 taxa), *Bornaviridae*: NP gene (JTT+Γ, 147 amino acids across 73 taxa), *Bornaviridae*: L gene (JTT+Γ+F, 1243 amino acids across 12 taxa), *Rhabdoviridae*: NP gene (LG+Γ, 220 amino acids across 34 taxa), *Rhabdoviridae*: L gene (LG+Γ+F, 383 amino acids across 26 taxa).

### Simulation

A Monte Carlo simulation procedure was employed to determine the probability that the bornavirus-derived element EBLN-1 has retained coding capacity over 54.1 million years under neutral evolution (i.e. not under purifying selection). A consensus EBLN-1 sequence was inferred, and the effects of neutral evolution were simulated using seq-gen [62] for a branch length equivalent to the minimum amount of time that EBLN-1 orthologs have resided in primate genomes, based on the primate divergences estimated by Bininda-Emonds *et al* [42], and given a neutral rate of evolution of 2.2×10–9 [12]. The number of stop codons accrued was counted for 100,000 iterations of the simulation. The probability that the reading frame could have remained open under neutrality is given by the number of replicates under which no stop codons have evolved, divided by the number of iterations.

### Sequences and accession numbers

*Parvoviridae*; AAV2 (NC_001401); Minute virus of mice (NC_001510.1); AMDV (NC_001662); Goose parvovirus (EU583390.1); Muscovy duck parvovirus (X75093.1); Porcine hokovirus (EU200671.1); Snake parvovirus (AY349010.1); Avian AAV (AY629582.1, AY629583.1, GQ368252.1); AAV1 (AF063497.1); AAV4 (U89790); AAV2 (AY695375.1); Bovine AAV (AY388617.1); Caprine AAV (DQ335246.2); Bocavirus (M14363.1); Erythrovirus (AB126265.1); Aleutian mink disease virus (M20036.1); Porcine parvovirus (EU790642.1); Feline panleukopenia virus (EF988660.1); Canine parvovirus (EU310373.2); Rat parvovirus (AF036710.1); Hamster parvovirus (U34255.1); Minute virus of mice (DQ196317.1); Kilham rat virus (U79033.1); *Circoviridae*; Porcine circovirus 1 (NC_006266); Porcine circovirus 2 (GU325757); Cyclovirus PK5006 (GQ404856.1); Cyclovirus NG14 (GQ404855.1); Human stool-associated circular virus NG13 (GQ404856.1); Beak and feather disease virus (AY450436.1); Columbid circovirus (AF252610.1); *Hepadnaviridae*; duck HBV (NC_001344); Stork HBV (AJ251937.1|); Heron HBV (NC_001486); Ross' Goose HBV (AY494849.1); Crane HBV (AJ441113.1); Sheldgoose HBV (AY494852.1); Snow goose HBV (AF111000.1); Woodchuck HBV (AF410861.1); *Flaviviridae*; Kamiti river virus (NC_005064); *Aedes* flavivirus (NC_012932); Quang binh virus (NC_012671); *Culex* flavivirus (NC_008604); Nakiwogo virus (GQ165809). *Reoviridae*; Liaoning virus (NC_007736 - NC_007747); Kadipiro virus (NC_004199, NC_004205-NC_004210, NC_004212-NC_004216); Banna virus (NC_004198, NC_004200-NC_004204, NC_004211, NC_004217-NC_004221). *Bunyaviridae*; Crimean-Congo hemorrhagic fever virus (NC_005300, NC_005301, NC_005302); Uukuniemi virus (NC_005214, NC_005220, NC_005221); Uukuniemi virus (M33551.1); Catch-me-cave virus (EU274384.1); Sandfly fever Naples virus (EF201832.1); Massilia virus (EU725773.1); Punta Toro virus (EF201834.1); Buenaventura virus (EF201839.1); Rift Valley fever virus (DQ380156.1); Phlebovirus sp. (EF201818.1); Icoaraci virus (EF076014.1). *Orthomyxoviridae*; Quaranfil virus (FJ861694.1); Johnston Atoll virus (FJ861696.1); Thogoto virus (M77280.1); Dhori virus (M34002.1). *Bornaviridae*; Borna disease virus (NC_001607); Avian BDV (FJ169441). *Filoviridae*; Reston ebola virus (NC_002549); Zaire ebola virus (NC_002549); Lake Victoria marburgvirus (NC_001608). *Rhabdoviridae*; vesicular stomatitis virus (NC_001560); Wongabel virus (NC_011639); Kotonkon virus (DQ457099); Adelaide river virus (U10363.1); Obodhiang virus (DQ457098.1); Bovine ephemeral fever virus (AF234533.1); Rochambeau virus (DQ457104.1); Mount elgon bat virus (DQ457103.1); Oita rhabdovirus (AB116386); Kern canyon virus (DQ457101.1); Sandjimba virus (DQ457102.1); Kolongo virus (DQ457100.1); Tupaia rhabdovirus (AY840978.1); Spring viremia of carp (DQ491000.1); Pike fry rhabdovirus (FJ872827.1); Cocal virus (EU373657.1); Vesicular stomatitis Indiana virus (AF473865.1); Isfahan virus (AJ810084.2); Chandipura virus (AY614728.1); Ngaingan virus (FJ715959.1); Wongabel virus (EF612701.1); Flanders virus (AF523194.1). *Nyaviridae*; Midway virus (NC_012702); Nyamanini virus (NC_012703).

## Supporting Information

Figure S1 Sequence alignment of an EVE identified in the *Aedes aegyptii* genome and Liaoning virus segment 5. Genomic regions, as determined by alignment to a repetitive element (RE) in the *A. aegyptii* genome, are indicated in blue, on coding viral regions are shown in red, and regions encoding viral proteins are shown in green.
(0.40 MB PDF)

Figure S2 Genetic structure of an adenovirus related sequence identified in whole-genome shotgun sequence data for Ord's kangaroo rat (*Dipodymys ordii*). The name of the corresponding protein in the most closely related virus (tree shrew adenovirus 1; AF258784.1) is indicated above each open reading frame (ORF). Arrows beneath ORFs indicate frames encoded in reverse direction relative to contig. Abbreviations: kd = kiloDalton; pol = DNA polymerase; T = terminal protein; P = penton base; Mco = minor core; Mca = minor capsid; DB = DNA binding; Ma = Major coat; H = hexon-associated; S = shaft.
(0.27 MB PDF)

Figure S3 Genetic structure of a complete dependovirus genome identified in the little brown bat (*Myotis lucifugus*) genome. The element is a composite of two genomic contigs, which were assembled by identifying the empty pre-integration site in the closest relative (*Pteropus vampyrus*). The inset box shows an alignment the inverted repeats in the 5′ and 3′ untranslated regions. Abbreviations: IR = inverted repeat. NS1 = Non-structural protein 1; VP2 = Viral protein 2; UTR = untranslated region.
(0.32 MB PDF)

Table S1 Genome sequences screened for endogenous viral elements.
(0.09 MB DOC)

Table S2 Viral reference sequences used for *in silico* screening of host genomes.
(0.14 MB DOC)

Table S3 Endogenous viral elements related to negative sense RNA viruses.
(0.40 MB DOC)

Table S4 Endogenous viral elements related to doubled-stranded RNA viruses.
(0.03 MB DOC)

Table S5 Endogenous viral elements related to positive sense RNA viruses.
(0.09 MB DOC)

Table S6 Endogenous viral elements related to single stranded DNA viruses.
(0.21 MB DOC)

Table S7 Endogenous viral elements related to reverse transcribing DNA viruses.
(0.04 MB DOC)
