# Supplementary material for: Endogenous Viral Elements in Animal Genomes
Source: PLoS Genet. 2010 Nov 18;6(11):e1001191. doi: 10.1371/journal.pgen.1001191 (PMC2987831; doi:10.1371/journal.pgen.1001191)
Supplement: Figure S1 — Sequence alignment of an EVE identified in the Aedes aegyptii genome and Liaoning virus segment 5. Genomic regions, as determined by alignment to a repetitive element (RE) in the A. aegyptii genome, are indicated in blue, on coding viral regions are shown in red, and regions encoding viral proteins are shown in green. (0.40 MB PDF) [file pgen.1001191.s001.pdf]

Figure S1

```
Liao ning virus segment 5 ---------- ---------- ---------- ---------- ---------- ----------
Aedes aegypti cont1.21    GGACAACCGT AGAGCAAAAG AAAAGGAACA ATAGGCACGA AGTGCAGGAG CGTAGGGATA
Aedes aegypti RE          GGACAACCGT AGTGAGAAAG AAGAAGAAGA AGAAACAGGA GAATCAGGAG CGCAGGGATA

Liao ning virus segment 5 ---------- ---------- ---------- ---------- ---------- ----------
Aedes aegypti cont1.21    CCAGGCCAAA GAAAGGTAGG AGGGAAGGTG CCAAGCGCGA AAAGGGTGAT GCGATCATCA
Aedes aegypti RE          CCAGGCCTAA GAAAGGTAGG AAGGTAGGTG CCAAGCGCGA AAAGGGTGAT GCGATCATCA

Liao ning virus segment 5 ---------- ---------- ------GTTA TTTTTATTCA ATGACTGTTT GAGCTACAAC
Aedes aegypti cont1.21    TCAAGACGGA AGAGTCCAAG TACTCG--.. .......... .A........ ..........
Aedes aegypti RE          TCAAGACGGA AGAGTCCAAG TACTCG---- ---------- ---------- ----------

Liao ning virus segment 5 CTGAACCTGC TTGACTAGAT TGCGCGGCGA TTTATTCAAC ACAAGCATTT ATCAGTACTT
Aedes aegypti cont1.21    .......... .......... .......A.. .......... .......... ........A.
Aedes aegypti RE          ---------- ---------- ---------- ---------- ---------- ----------

Liao ning virus segment 5 AGTTTACATC AATCGCAACT TATAACCATA GAACAAGTAT TTTGATTTAT TCCATTATAT
Aedes aegypti cont1.21    ..A.C..... ..C.....A. ..A..T.G.. ..TT....T. .....C.... .......C..
Aedes aegypti RE          ---------- ---------- ---------- ---------- ---------- ----------

Liao ning virus segment 5 TTACATATAT TACCGTGACT TTGGGAA-GA TTACTAGTTA TTAAGTATAT ACACCATAAT
Aedes aegypti cont1.21    .......... .......... .......AAC ..GT...CC. ....T..... .T.TT...T.
Aedes aegypti RE          ---------- ---------- -------.-- ---------- ---------- ----------

Liao ning virus segment 5 TGAGGATTAT TTACTTTAAG TCATAC-TTA CTTAATCTCT TCATTTACAT CGATTTTAAC
Aedes aegypti cont1.21    .T........ ....C..... A.G...ACC. .....C..T. AAT..GT.TA ACCG.C.G..
Aedes aegypti RE          ---------- ---------- ------.--- ---------- ---------- ----------

Liao ning virus segment 5 CGTTCTCAAA ATGTACCAGC AAATTGACTT CAACGCAGTT GATTACAGTG TGCTCTACAA
Aedes aegypti cont1.21    ......TG.. .A......A. .G........ ......CA.. .......... .A..T.....
Aedes aegypti RE          ---------- ---------- ---------- ---------- ---------- ----------

Liao ning virus segment 5 TGCAGGAGAG CAGTTGGTTC AACAAACCGC CATTGTTGGA CCACCTACCA ACTATGAAGC
Aedes aegypti cont1.21    ....A.T.GA ........C. GG.C...... .......... ......G.TG C..TC.....
Aedes aegypti RE          ---------- ---------- ---------- ---------- ---------- ----------

Liao ning virus segment 5 AATTTATTCC ACACCGCTAA TGCCTTACGT TGCTGTTCCG GCCGTTACTT CAATGATGGA
Aedes aegypti cont1.21    C......C.T ..G...A.GG ........-- ---------- ---------- ----------
Aedes aegypti RE          ---------- ---------- ---------- ---------- ---------- ----------

Liao ning virus segment 5 TATACACCTA ATGAGCCATC TCTCGGTTAA CGTTACAGGA ACTAATCTGG CAAGATACCA
Aedes aegypti cont1.21    ---------- ---------- ---------- ---------- ------G.C. .......T..
Aedes aegypti RE          ---------- ---------- ---------- ---------- ---------- ----------

Liao ning virus segment 5 CGTCGGTAAC TCTTGTCGTA TCACAGAAAG TTCAGTTCTT GAGTTGCCTG TTGGTGACGA
Aedes aegypti cont1.21    ...T.....T ..C.....C. .A..G..... ...GA..... ........C. .A..C..T..
Aedes aegypti RE          ---------- ---------- ---------- ---------- ---------- ----------

Liao ning virus segment 5 GACCCGTTAC TTCAAGCCAC TCAAGTGTTC TGGACCATAT GCAATATTTG AACGTCTTCC
Aedes aegypti cont1.21    ......A..T ........GT .G.....C.. G.....G..C ........C. ....C..A..
Aedes aegypti RE          ---------- ---------- ---------- ---------- ---------- ----------

Liao ning virus segment 5 ATGTGAAAAG GACTACCGTG TCAACGGGCT CCTTCATCAC TTTGGAGTCG TTCCGATTAC
Aedes aegypti cont1.21    G.....G..T ..T.....A. .T...A.... A..G..C... ........G. .....G....
Aedes aegypti RE          ---------- ---------- ---------- ---------- ---------- ----------

Liao ning virus segment 5 ATTAGTCCGT GACATAGCCG AGATTGAGCC CATTATGGAG GACTACTTCA TTAGTGGCCC
Aedes aegypti cont1.21    TC........ .......TT. .A..C..... .........A .......... .......T..
Aedes aegypti RE          ---------- ---------- ---------- ---------- ---------- ----------

Liao ning virus segment 5 AGTCGGACCG TCGATCACAT GTTTAATGAA AACGTATGCC TTAAGACAAG TAGAGATAGT
Aedes aegypti cont1.21    C..T...... ..A.....T. .C..G..... .......... .......... .G.....T..
Aedes aegypti RE          ---------- ---------- ---------- ---------- ---------- ----------

Liao ning virus segment 5 GTTCGGAGAA AAGGGATTGT ACGTCAGACC CACCGGGTCC AAGGATCTGT CAAAGACAGA
Aedes aegypti cont1.21    A..T.....T ..A....... ..A.T..... ...T...... ......T.T. .T..A.TT..
Aedes aegypti RE          ---------- ---------- ---------- ---------- ---------- ----------

Liao ning virus segment 5 AGACTGCTGT CGTCAAGCCT TTGCCTTAAA CAATGAACGC ACAAGATGCC ATAAACAACT
Aedes aegypti cont1.21    T..T..T... ...A.T.... .......G.G .........A ..C....... ..........
Aedes aegypti RE          ---------- ---------- ---------- ---------- ---------- ----------

Liao ning virus segment 5 TAAACCAGAC AAACGTACTA AGAAATCTAC CTCCAAGGAT ATAAAGAAAC AGAACAAAGT
Aedes aegypti cont1.21    .........T .....CGA.. .....GG.G. ......AATA T...GA..CA G.T.T..CT.
Aedes aegypti RE          ---------- ---------- ---------- ---------- ---------- ----------

Liao ning virus segment 5 CGTTGACCCC AAGGTGTCGA ACCACGGTCA TTAA-CCAAA GATTCGGAA- TAACATCAAA
Aedes aegypti cont1.21    A...A..... .......... ....T...TG G....-ACT. ATC....... ..GT......
Aedes aegypti RE          ------???? ?????????? ?????????? ?????????? -T.G....TC ..GG.G.CG.

Liao ning virus segment 5 ACAGGGAACT ATGCTATAAC ATCATACTAA A-GCTTTGCA ATCAAGTCAG GATGTGAGTC
Aedes aegypti cont1.21    .TT....... .C.T...... ...G...... .AA..A..AT .......... ..........
Aedes aegypti RE          CGTAC.C.G. G.CAG.CGCA C..G...--- -.---AG.TG .AATGA..CT CGA.CTTAAG

Liao ning virus segment 5 TCAGACCGCA TGCTTTAAGT CCGCGAATAA CCCGAC---- ---------- ----------
Aedes aegypti cont1.21    ...A...... .......... ..A.TCGG.. GT.TTGAAGG CGATGC..GC AGCGACGCGA
Aedes aegypti RE          CGC..TAAGG A..GCA.G.G .GC..CC..C AAAAGTTTGG CGGAAGAGGT CCTTGGCGCT

Liao ning virus segment 5 ---------- ---------- ---------- ---------- ---------- ----------
Aedes aegypti cont1.21    AGCTCATAGA TCTACGATCG TACGCAGTGT CAGAGGCATT GGCTCTGAAG GTGATGAATC
Aedes aegypti RE          GGTGTTGAAG TGAGGGCTCT GACGCAGTCA GTGA...... ..CTCTGAAG GTGATGAACC

Liao ning virus segment 5 ---------- ---------- ---------- ---------- ---------- ----------
Aedes aegypti cont1.21    TTGACGAGAT CACTGACGCA GAAGAGCTCG TCACGGCACT GCG....... ....AGGTAC
Aedes aegypti RE          TTGACGAGAT CACCAACGCA GAAGAGCTCG TCACGGCACT GCGGCAACAG TGCGAAGTGC
```
