# Supplementary figures and images for: Endogenous Viral Elements in Animal Genomes
Source: PLoS Genet. 2010 Nov 18;6(11):e1001191. doi: 10.1371/journal.pgen.1001191 (PMC2987831; doi:10.1371/journal.pgen.1001191)

Figure S2

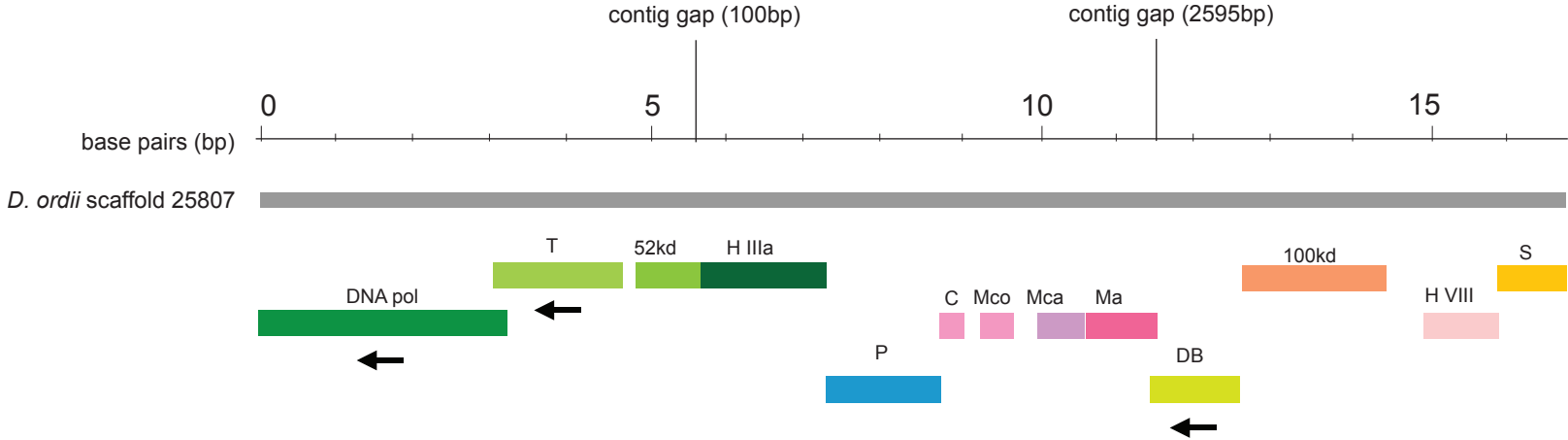

Supplement: Figure S2 — Genetic structure of an adenovirus related sequence identified in whole-genome shotgun sequence data for Ord's kangaroo rat (Dipodymys ordii). The name of the corresponding protein in the most closely related virus (tree shrew adenovirus 1; AF258784.1) is indicated above each open reading frame (ORF). Arrows beneath ORFs indicate frames encoded in reverse direction relative to contig. Abbreviations: kd = kiloDalton; pol = DNA polymerase; T = terminal protein; P = penton base; Mco = minor core; Mca = minor capsid; DB = DNA binding; Ma = Major coat; H = hexon-associated; S = shaft. (0.27 MB PDF) [file pgen.1001191.s002.pdf]

Figure S3

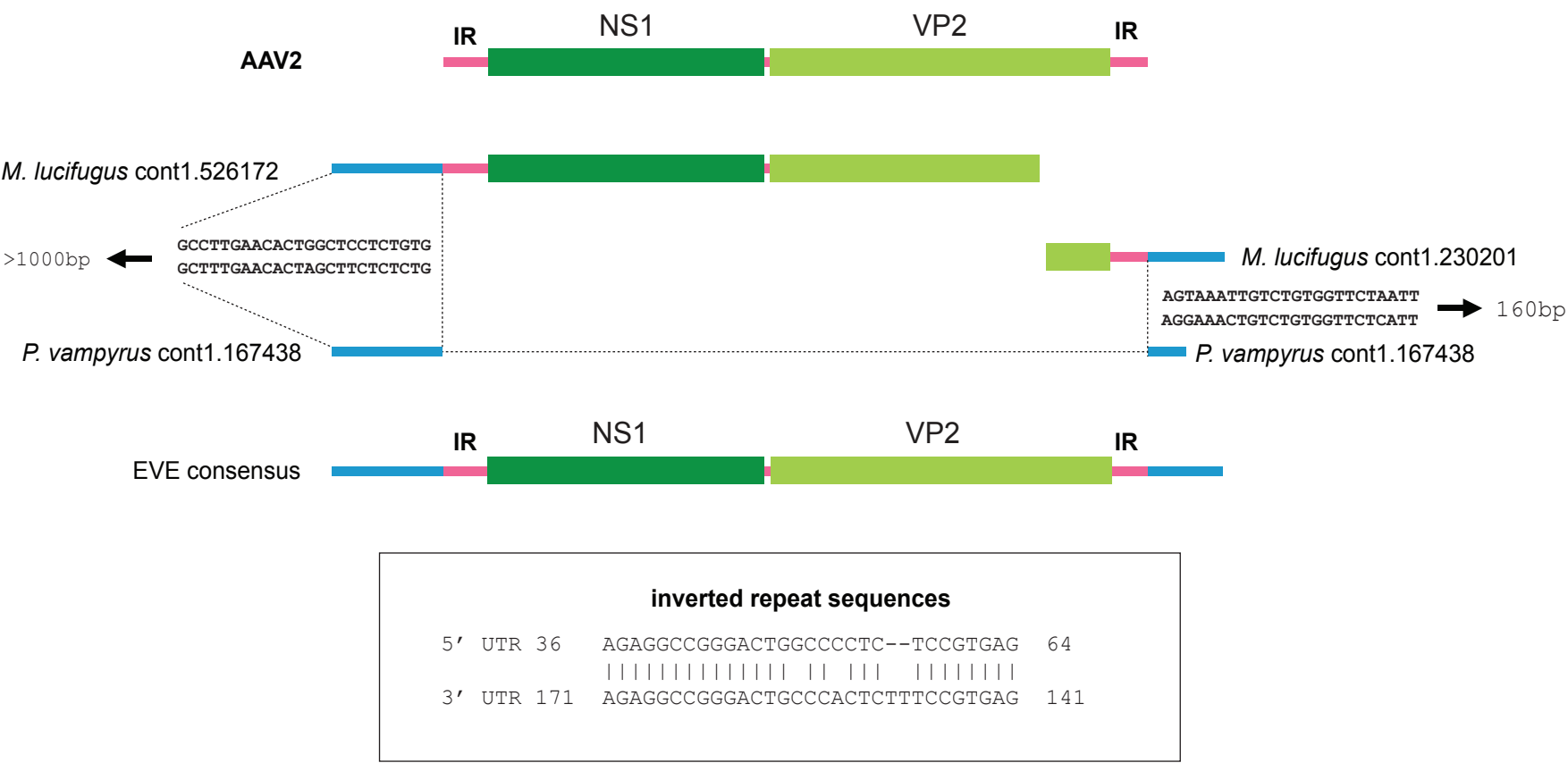

Supplement: Figure S3 — Genetic structure of a complete dependovirus genome identified in the little brown bat (Myotis lucifugus) genome. The element is a composite of two genomic contigs, which were assembled by identifying the empty pre-integration site in the closest relative (Pteropus vampyrus). The inset box shows an alignment the inverted repeats in the 5′ and 3′ untranslated regions. Abbreviations: IR = inverted repeat. NS1 = Non-structural protein 1; VP2 = Viral protein 2; UTR = untranslated region. (0.32 MB PDF) [file pgen.1001191.s003.pdf]
