# Supplementary material for: Endogenous Viral Elements in Animal Genomes
Source: PLoS Genet. 2010 Nov 18;6(11):e1001191. doi: 10.1371/journal.pgen.1001191 (PMC2987831; doi:10.1371/journal.pgen.1001191)
Supplement: Table S1 — Genome sequences screened for endogenous viral elements. (0.09 MB DOC) [file pgen.1001191.s004.doc]

**Table S1.** Genome sequences screened for endogenous viral elements

| Species name | Common name | Order | Version |
| --- | --- | --- | --- |
| Class Aves | | | |
| *Gallus gallus* | Chicken | Galliformes | NCBI 2.1 November 2006 |
| *Taeniopygia guttata* | Zebrafinch | Passeriformes | NCBI 1.1 March 2009 |
| Class Mammalia | | | |
| *Microcebus murinus* | Grey mouse lemur | Primates | EMBL 1.54 June 2009 |
| *Tarsius syrichta* | Tarsier | Primates | EMBL 1.54 June 2009 |
| *Otolemur garnettii* | Bush baby | Primates | EMBL 1.54 June 2009 |
| *Callithrix jacchus* | Common marmoset | Primates | WUSTL 1.0 2009 |
| *Macaca mulatta* | Rhesus macaque | Primates | NCBI 1.1 June 2006 |
| *Papio hamadryas* | Hamadryas baboon | Primates | Baylor 1.0 June 2009 |
| *Pongo pygmaeus* | Orang-utan | Primates | EMBL 2.54 June 2009 |
| *Gorilla gorilla* | Gorilla | Primates | EMBL 1.54 June 2009 |
| *Pan troglodytes* | Chimpanzee | Primates | NCBI 2.1 October 2006 |
| *Homo sapiens* | Human | Primates | NCBI 36.3 March 2008 |
| *Vicugna pacos* | Alpaca | Artiodactyla | EMBL 1.54 June 2009 |
| *Bos taurus* | Cow | Artiodactyla | NCBI 4.1 August 2008 |
| *Sus scrofa* | Pig | Artiodactyla | NCBI 1.1 July 2008 |
| *Equus caballus* | Horse | Perissodactyla | NCBI 2.1 July 2008 |
| *Ailuropoda melanoleuca* | Panda | Carnivora | BGI Feb 2010 |
| *Canis familiaris* | Domestic dog | Carnivora | NCBI 2.1 September 2005 |
| *Felis catus* | Domestic cat | Carnivora | EMBL 1.54 June 2009 |
| *Sorex araneus* | Common shrew | Soricomorpha | EMBL 1.54 June 2009 |
| *Pteropus vampyrus* | Flying fox | Chiroptera | EMBL 1.54 June 2009 |
| *Myotis lucifugus* | Little brown bat | Chiroptera | EMBL 1.54 June 2009 |
| *Cavia porcellus* | Guinea pig | Rodents | EMBL 3.54 June 2009 |
| *Spermophilus tridecemlineatus* | Thirteen lined ground squirrel | Rodents | EMBL 1.54 June 2009 |
| *Mus musculus* | Mouse | Rodents | NCBI 37.1 July 2007 |
| *Rattus norvegicus* | Brown rat | Rodents | NCBI 4.1 June 2006 |
| *Dipodomys ordii* | Kangaroo rat | Rodents | EMBL 1.54 June 2009 |
| *Ochotona princeps* | Pika | Lagomorpha | EMBL 1.54 June 2009 |
| *Oryctolagus cuniculus* | European rabbit | Lagomorpha | EMBL 1.54 June 2009 |
| *Loxodonta africana* | African elephant | Proboscidea | EMBL 2.54 June 2009 |
| *Tursiops truncatus* | Bottlenose dolphin | Cetacea | Baylor 1.0 June 2009 |
| *Procavia capensis* | Cape hyrax | Hyracoidea | EMBL 1.54 June 2009 |
| *Erinaceus europaeus* | European hedgehog | Erinaceomorpha | EMBL 1.54 June 2009 |
| *Echinops telfari* | Lesser hedgehog tenrec | Afrosoricida | EMBL 1.54 June 2009 |
| *Tupaia belangeri* | Tree shrew | Scandentia | EMBL 1.54 June 2009 |
| *Choloepus hoffmanni* | Hoffmann’s three toed sloth | Pilosa | EMBL 1.54 June 2009 |
| *Dasypus novemcinctus* | Nine-banded armadillo | Cingulata | EMBL 2.54 June 2009 |
| *Monodelphis domestica* | Oppossum | Didelphimorphia | NCBI 2.1 March 2007 |
| *Macropus eugenii* | Tammar wallaby | Diprotodontia | Baylor 1.1 June 2009 |
| *Ornithorhynchus anatinus* | Duck-billed platypus | Monotremata | NCBI 1.1 July 2007 |
| Class Arthropoda | | | |
| *Ixodes scapularis* | Black-legged tick | Ixodida | VectorBase IscaW1 |
| *Aedes aegypti* | Yellow fever mosquito | Diptera | VectorBase AaegL1 |
| *Anopheles gambiae* |  | Diptera | VectorBase AgamP3 |
| *Culex quinquefasciatus* | Southern house mosquito | Diptera | VectorBase, Celera CpipJ1 |
|  |  |  |  |
