# Supplementary material for: Endogenous Viral Elements in Animal Genomes
Source: PLoS Genet. 2010 Nov 18;6(11):e1001191. doi: 10.1371/journal.pgen.1001191 (PMC2987831; doi:10.1371/journal.pgen.1001191)
Supplement: Table S2 — Viral reference sequences used for in silico screening of host genomes. (0.14 MB DOC) [file pgen.1001191.s005.doc]

**Table S2. Viral reference sequences used for *in silico* screening of host genomes.**

| **Family** | **Genus** | **Type species** | **Accession**  **numbers** |
| --- | --- | --- | --- |
| **RNA-ve** | | | |
| *Arenaviridae* | Arenavirus | Lymphocytic choriomeningitis virus | NC_004291  NC_004294 |
| *Bornaviridae* | Bornavirus | Borna disease virus | NC_001607 |
| *Bunyaviridae* | Orthobunyavirus | Bunyamwera virus | NC_001925  NC_001926  NC_001927 |
| *Bunyaviridae* | Hantavirus | Hantaan virus | NC_005218  NC_005219  NC_005222 |
| *Bunyaviridae* | Phlebovirus | Rift Valley fever virus | NC_002043  NC_002044  NC_002045 |
| *Filoviridae* | Marburgvirus | Lake Victoria marburgvirus | NC_001608 |
| *Filoviridae* | Ebolavirus | Zaire ebolavirus | NC_002549 |
| *Orthomyxoviridae* | Influenzavirus A | Influenza A virus | NC_007366  NC_007367  NC_007368  NC_007369  NC_007370  NC_007371  NC_007372  NC_007373 |
| *Orthomyxoviridae* | Thogotovirus | Thogoto virus | NC_006508  NC_006495  NC_006496  NC_006506  NC_006507  NC_006504 |
| *Paramyxoviridae* | Rubulavirus | Mumps virus | NC_002200 |
| *Paramyxoviridae* | Avulavirus | Newcastle disease virus | NC_002617 |
| *Paramyxoviridae* | Respirovirus | Sendai virus | NC_001552 |
| *Paramyxoviridae* | Henipavirus | Hendra virus | NC_001906 |
| *Paramyxoviridae* | Morbillivirus | Measles virus | NC_001498 |
| *Paramyxoviridae* | Pneumovirus | Human respiratory syncytial virus | NC_001781 |
| *Rhabdoviridae* | Vesiculovirus | Vesicular stomatitis Indiana virus | NC_001560 |
| *Rhabdoviridae* | Ephemerovirus | Bovine ephemeral fever virus | NC_002526 |
| *Rhabdoviridae* | Novirhabdovirus | Infectious hematopoietic necrosis virus | NC_001652 |
| *Unassigned* | Deltavirus | Hepatitis delta virus | NC_001653 |
| **RNA+ve** | | | |
| *Arteriviridae* | Arterivirus | Equine arteritis virus | NC_002532 |
| *Astroviridae* | Avastrovirus | Turkey astrovirus | NC_002470 |
| *Astroviridae* | Mamastrovirus | Human astrovirus | NC_001943 |
| *Caliciviridae* | Lagovirus | Rabbit hemorrhagic disease virus | NC_001543 |
| *Caliciviridae* | Norovirus | Norwalk virus | NC_001959 |
| *Caliciviridae* | Sapovirus | Sapporo virus | NC_006554 |
| *Caliciviridae* | Vesivirus | Vesicular exanthema of swine virus | NC_002551 |
| *Coronaviridae* | Coronavirus | Infectious bronchitis virus | NC_001451 |
| *Flaviviridae* | Flavivirus | Yellow fever virus | NC_002031 |
| *Flaviviridae* | Pestivirus | Bovine viral diarrhea virus 1 | NC_001461 |
| *Flaviviridae* | Hepacivirus | Hepatitis C virus | NC_004102 |
| *Picornaviridae* | Enterovirus | Poliovirus | NC_002058 |
| *Picornaviridae* | Rhinovirus | Human rhinovirus A | NC_001617 |
| *Picornaviridae* | Cardiovirus | Encephalomyocarditis virus | NC_001479 |
| *Picornaviridae* | Aphthovirus | Foot-and-mouth disease virus | NC_011450 |
| *Picornaviridae* | Hepatovirus | Hepatitis A virus | NC_001489 |
| *Picornaviridae* | Parechovirus | Human parechovirus | NC_001897 |
| *Picornaviridae* | Erbovirus | Equine rhinitis B virus | NC_003983 |
| *Picornaviridae* | Kobuvirus | Aichi virus | NC_001918 |
| *Picornaviridae* | Teschovirus | Porcine teschovirus | NC_003985 |
| *Togaviridae* | Alphavirus | Sindbis virus | NC_001547 |
| *Togaviridae* | Rubivirus | Rubella virus | NC_001545 |

**Table S2. Viral reference sequences used for *in silico* screening of host genomes (continued).**

| **Family** | **Genus** | **Type species** | **Accession**  **numbers** |
| --- | --- | --- | --- |
| **RNA-ve (continued)** |  |  |  |
| Unassigned | Hepevirus | Hepatitis E virus | NC_001434 |
| *Nodaviridae* | Alphanodavirus | Nodamura virus | NC_002690 |
|  |  |  |  |
| **dsRNA** |  |  |  |
| *Birnaviridae* | Avibirnavirus | Infectious bursal disease virus | NC_004178  NC_004179 |
| *Reoviridae* | Orthoreovirus | Mammalian orthoreovirus | NC_004271  NC_004259  NC_004255  NC_004261  NC_004262  NC_004257  NC_004267  NC_004268  NC_004266  NC_004265 |
| *Reoviridae* | Orbivirus | Bluetongue virus | NC_006023  NC_006015  NC_006013  NC_006014  NC_006024  NC_006025  NC_006010  NC_006022  NC_006007  NC_006008 |
| *Reoviridae* | Rotavirus | Rotavirus A | NC_011507  NC_011504  NC_011505  NC_011506  NC_011508  NC_011510  NC_011500  NC_011509  NC_011502  NC_011501  NC_011503 |
| *Reoviridae* | Coltivirus | Colorado tick fever virus | NC_004181  NC_004189  NC_004191  NC_004190  NC_004182  NC_004183  NC_004184  NC_004185  NC_004186  NC_004187  NC_004188  NC_004180 |

**Table S2. Viral reference sequences used for *in silico* screening of host genomes (continued).**

| **Family** | **Genus** | **Type species** | **Accession**  **numbers** |
| --- | --- | --- | --- |
| dsDNA-RT | | | |
| *Hepadnaviridae* | Orthohepadnavirus | Hepatitis B virus | NC_003977 |
| *Hepadnaviridae* | Avihepadnavirus | Duck hepatitis B virus | NC_001344 |
| ssDNA | | | |
| *Circoviridae* | Circovirus | Porcine circovirus 1 | NC_006266 |
| *Circoviridae* | Gyrovirus | Chicken anemia virus | M55918 |
| *Parvoviridae* | Parvovirus | Minute virus of mice | NC_001510 |
| *Parvoviridae* | Erythrovirus | Human parvovirus B19 | NC_000883 |
| *Parvoviridae* | Dependovirus | Adeno-associated virus-2 | NC_001401.2 |
| *Parvoviridae* | Amdovirus | Aleutian mink disease virus | NC_001662 |
| *Parvoviridae* | Bocavirus | Bovine parvovirus | NC_001540 |
| *Anelloviridae* | Alphatorquevirus | Torque teno virus 1 | AB041007 |
| *Anelloviridae* | Betatorquevirus | Torque teno mini virus 1 | AB026931 |
| *Anelloviridae* | Gammatorquevirus | Torque teno midi virus 1 | AB290918 |
| *Anelloviridae* | Deltatorquevirus | Torque teno tupaia virus | AB057358 |
| *Anelloviridae* | Epsilontorquevirus | Torque teno tamarin virus | AB041960 |
| *Anelloviridae* | Zetatorquevirus | Torque teno douroucouli virus | AB041961 |
| *Anelloviridae* | Etatorquevirus | Torque teno felis virus | AB076003 |
| *Anelloviridae* | Thetatorquevirus | Torque teno canis virus | AB076002 |
| *Anelloviridae* | Iotatorquevirus | Torque teno sus virus 1 | AB076001 |
| dsDNA | | | |
| *Adenoviridae* | Mastadenovirus | Human adenovirus C | NC_001405 |
| *Adenoviridae* | Aviadenovirus | Fowl adenovirus A | NC_001720 |
| *Adenoviridae* | Atadenovirus | Ovine adenovirus D | NC_004037 |
| *Adenoviridae* | Siadenovirus | Turkey adenovirus A | NC_001958 |
| *Papillomaviridae* | Alphapapillomavirus | Human papillomavirus type 32 | NC_001586 |
| *Papillomaviridae* | Betapapillomavirus | Human papillomavirus type 5 | NC_001531 |
| *Papillomaviridae* | Gammapapillomavirus | Human papillomavirus 4 | NC_001457 |
| *Papillomaviridae* | Deltapapillomavirus | European elk papillomavirus | NC_001524 |
| *Papillomaviridae* | Epsilonpapillomavirus | Bovine papillomavirus 5 | NC_004195 |
| *Papillomaviridae* | Etapapillomavirus | Fringilla coelebs papillomavirus | NC_004068 |
| *Papillomaviridae* | Iotapapillomavirus | *Mastomys natalensis* papillomavirus | NC_001605 |
| *Papillomaviridae* | Kappapapillomavirus | Cottontail rabbit papillomavirus | NC_001541 |
| *Papillomaviridae* | Lambdapapillomavirus | Canine oral papillomavirus | NC_001619 |
| *Papillomaviridae* | Mupapillomavirus | Human papillomavirus 1 | NC_001356 |
| *Papillomaviridae* | Nupapillomavirus | Human papillomavirus type 41 | NC_001354 |
| *Papillomaviridae* | Omikronpapillomavirus | *Phocoena spinipinnis* papillomavirus | NC_003348 |
| *Papillomaviridae* | Pipapillomavirus | *Rattus norvegicus* papillomavirus 1 | NC_003348 |
| *Papillomaviridae* | Thetapapillomavirus | *Psittacus erithacus timneh* papillomavirus | NC_003973 |
| *Papillomaviridae* | Xipapillomavirus | Bovine papillomavirus 3 | NC_004197.1 |
| *Papillomaviridae* | Zetapapillomavirus | Equine papillomavirus 1 | NC_003748 |
| *Polyomaviridae* | Polyomavirus | Simian virus 40 | NC_001669 |
