# Supplementary material for: Endogenous Viral Elements in Animal Genomes
Source: PLoS Genet. 2010 Nov 18;6(11):e1001191. doi: 10.1371/journal.pgen.1001191 (PMC2987831; doi:10.1371/journal.pgen.1001191)
Supplement: Table S3 — Endogenous viral elements related to negative sense RNA viruses. (0.40 MB DOC) [file pgen.1001191.s006.doc]

**Table S3.** Endogenous viral elements related to negative sense RNA viruses

| Host species ^1^ | Contig ^2^ | Location ^3^ | 4 | Best viral match ^5^ | NR  e-value ^6^ | PFAM  e-value ^7^ | Genomic  region ^8^ | Element name ^9^ |
| --- | --- | --- | --- | --- | --- | --- | --- | --- |
| ***Bornaviridae*** |  |  |  |  |  |  |  |  |
| *Bornavirus* |  |  |  |  |  |  | Borna disease virus |  |
| Grey mouse lemur | ABDC01288276 | 15251-16019 | - | FJ169440 | 1e-31 | 1.7e-29 | 78-839 |  |
| (*Microcebus murinus*) | ABDC01288276 | 14606-14946 | - | FJ169441 | 2e-13 | N/A | 1893-2261 |  |
| Small-eared galago (*Otolemur garnetti*) | AAQR01574020 | 2755-3258 | - | FJ792853 | 2e-17 | 1.2e-18 | 534-1022 |  |
| Common marmoset [16] | ACFV01032871 | 23431-24116 | + | AJ311524 | 6e-45 | 1.1e-48 | 435-1100 | EBLN-1 (NP) |
| (*Callithrix jacchus*) | ACFV01016030 | 26616-27000 | + | FJ169440 | 9e-38 | 1.2e-35 | 135-1046 | EBLN-2 (NP) |
|  | ACFV01189008 | 2214-3596 | - | AY374546 | 4e-36 | 1.6e-28 | 93-1157 | EBLN-4 (NP) |
|  | ACFV01033973 | 90483-91260 | + | AY374546 | 4e-36 | 3.8e-50 | 171-965 | EBLN-3 (NP) |
|  | ACFV01099682 | 6640-7739 | - | AJ311524 | 3e-58 | 4e-67 | 138-1166 |  |
|  | ACFV01001945 | 16973-18042 | - | AY374525 | 8e-55 | 1.3e-55 | 156-1166 |  |
|  | ACFV01155689 | 91-836 | - | AJ311524 | 4e-30 | 1.8e-36 | 138-848 |  |
|  | ACFV01177522 | 2-736 | + | AY374542 | 5e-27 | 4.3e-30 | 345-1094 |  |
|  | ACFV01193145 | 1972-2655 | - | EU095836 | 43-26 | 1.5e-24 | 444-1094 |  |
|  | ACFV01177817 | 6612-7256 | - | AY374542 | 3e-25 | 6.3e-26 | 408-1046 |  |
|  | ACFV01186132 | 4614-5258 | + | DQ680833 | 8e-24 | 1.6e-24 | 444-1094 |  |
|  | ACFV01177095 | 14919-15666 | + | AY374542 | 4e-24 | 1.3e-20 | 444-1094 |  |
|  | ACFV01177695 | 7386-7958 | - | EU095836 | 6e-23 | 4.2e-27 | 333-920 |  |
|  | ACFV01016031 | 101-677 | + | FJ169441 | 1e-24 | 1.6e-29 | 513-1106 |  |
|  | ACFV01137553 | 1602-2060 | - | AB032031 | 4e-11 | 1.8e-14 | 2314-2700 | EBLN-5 (GP) |
| Gorilla | CABD02038126 | 6170-7273 | - | FJ169441 | 3e-70 | 1.6e-79 | 135-1166 | EBLN-1 (NP) |
| (*Gorilla gorilla*) | CABD02246393 | 3733-4740 | + | FJ169440 | 1e-52 | 7.7e-46 | 135-1046 | EBLN-2 (NP) |
|  | CABD02145130 | 344-1421 | + | FJ169440 | 2e-51 | 1.3e-49 | 93-1157 | EBLN-4 (NP) |
|  | CABD02377713 | 5487-6564 | + | AY374519 | 2e-51 | 2.9e-48 | 96-1046 | EBLN-3 (NP) |
|  | CABD02009223 | 226-311 | - | AF136236 | 3e-08 | 8.5e-20 | 2305-2613 | EBLN-5 (GP) |
| Human | NC_000010.10 | 22497812-22498912 | - | FJ169441 | 3e-72 | 4e-77 | 135-1166 | EBLN-1 (NP) |
| (*Homo sapiens*) | NC_000003.11 | 73111374-73112327 | + | FJ169441 | 4e-53 | 3.6e-46 | 135-1046 | EBLN-2 (NP) |
|  | NC_000017.10 | 4474361-4475442 | + | FJ169440 | 2e-49 | 1.3e-44 | 93-1157 | EBLN-4 (NP) |
|  | NC_000009.11 | 37086724-37087800 | + | FJ169440 | 3e-53 | 5.5e-53 | 96-1157 | EBLN-3 (NP) |
|  | NC_000001 | 4596986-45969751 | - | BDU94866 | 6e-10 | 1.7e-20 | 2251-2613 | EBLG-5 (GP) |
| Chimpanzee | NC_006477 | 22753238-22754341 | - | FJ169441 | 4e-72 | 1.7e-80 | 135-1166 | EBLN-1 (NP) |
| (*Pan troglodytes*) | NC_006490 | 74962397-74963472 | + | FJ620690 | 1e-52 | 1.3e-40 | 135-1046 | EBLN-2 (NP) |
|  | NC_006484 | 4658675-4659758 | + | FJ169440 | 2e-49 | 5.1e-47 | 93-1157 | EBLN-4 (NP) |
|  | NC_006476 | 37852382-37853459 | + | AY374519 | 2e-54 | 1e-53 | 93-1157 | EBLN-3 (NP) |
|  | NC_006468 | 46259800-46260265 | - | BDU94866 | 2e-10 | 2.3e-21 | 2251-2613 | EBLG-5 (GP) |
| Orang utan | ABGA01377867 | 11377-12480 | - | FJ169441 | 3e-75 | 3.7e-84 | 135-1166 | EBLN-1 (NP) |
| (*Pongo abelii*) | ABGA01086692 | 1783-2787 | - | AJ311524 | 4e-48 | 6.4e-44 | 87-1046 | EBLN-2 (NP) |
|  | ABGA01196413 | 3791-4873 | - | FJ169440 | 2e-52 | 1.1e-48 | 93-1157 | EBLN-4 (NP) |
|  | ABGA01388722 | 1609-525 | - | AY374519 | 1e-56 | 4e-58 | 93-1157 | EBLN-3 (NP) |
|  | ABGA01395387 | 2684-3143 | + | BDU94874 | 6e-10 | 5e-18 | 2251-2652 | EBLG-5 (GP) |
| White-cheeked gibbon | *N/A* | *N/A* | *N/A* | EU781967 | 2e-51 | 8.8e-76 | 135-1100 | EBLN-1 (NP) |
| (*Nomascus leucogenys*) | *N/A* | *N/A* | *N/A* | AJ311524 | 5e-68 | 1.7e-44 | 135-1046 | EBLN-2 (NP) |
|  | *N/A* | *N/A* | *N/A* | FJ169440 | 5e-50 | 4.9e-44 | 93-1157 | EBLN-4 (NP) |
|  | *N/A* | *N/A* | *N/A* | AY374519 | 5e-55 | 1.3e-53 | 96-1157 | EBLN-3 (NP) |
|  | *N/A* | *N/A* | *N/A* | BDU94874 | 3e-09 | 9.2e-20 | 2251-2607 | EBLG-5 (GP) |
| Rhesus macaque | NC_007866 | 22405827-22406447 | - | FJ169441 | 1e-69 | 2.6e-77 | 135-1100 | EBLN-1 (NP) |
| (*Macaca mulatta*) | NC_007859 | 63156561-63157517 | - | EU781967 | 7e-57 | 1.8e-46 | 87-1046 | EBLN-2 (NP) |
|  | NC_007873 | 4346813-4347846 | + | FJ169440 | 7e-53 | 2.5e-48 | 105-1157 | EBLN-4 (NP) |
|  | NC_007872 | 40346300-40347161 | - | AY374519 | 2e-54 | 2.9e-52 | 96-956 | EBLN-3 (NP) |
|  | NC_007858 | 48422406-48422783 | - | AJ311524 | 3e-08 | 1.8e-46 | 2251-2700 | EBLG-5 (GP) |
| Hamadyras baboon | 469538 | 12188-12823 | + | FJ169441 | 4e-51 | 3e-59 | 135-890* | EBLN-1 (NP) |
| (*Papio hamadryas*) | 7504_471868  638211_352285 | 216337-217277 | + | EU781967 | 2e-59 | 2.8e-48 | 87-1046 | EBLN-2 (NP) |
|  | 352639_745223 | 71183-71887 | - | FJ169440 | 1e-50 | 1.2e-47 | 93-1157 | EBLN-4 (NP) |
|  | 264969_515420 | 161428-162428 | + | FJ169440 | 4e-52 | 4.1e-56 | 96-1157 | EBLN-3 (NP) |

**Table S3.** Endogenous viral elements related to negative sense RNA viruses (continued)

| Host species ^1^ | Contig ^2^ | Location ^3^ | 4 | Best viral match ^5^ | NR  e-value ^6^ | PFAM  e-value ^7^ | Genomic  region ^8^ | Element name ^9^ |
| --- | --- | --- | --- | --- | --- | --- | --- | --- |
| ***Bornaviridae (continued)*** |  |  |  |  |  |  |  |  |
| *Bornavirus* |  |  |  |  |  |  | Borna disease virus |  |
| African Elephant [14] | AAGU03015682 | 8568-9689 | - | AY374520 | 2e-25 | 1.4e-32 | 360-1127 | EBLN-6 (NP) |
| (*Loxodonta African*) | AAGU03024746 | 17448-18540 | + | DQ680833 | 2e-16 | 9.6e-23 | 633-1019 | EBLN-6 (NP) |
|  | AAGU03049012 | 9142-9474  14966-15685 | - | DQ680833 | 2e-16 | 9.7e-23 | 633-1019 | EBLN-6 (NP) |
|  | AAGU03021424 | 11188-12264 | - | DQ680833 | 4e-12 | 6.3e-13 | 477-1040 | EBLN-6 (NP) |
|  | AAGU03043349 | 106085-107223 | - | FJ169441 | 4e-39 | 1.1e-32 | 291-1037 |  |
|  | AAGU03027606 | 35816 -36577 | + | FJ620690 | 2e-37 | 1.7e-37 | 258-1046 |  |
| Cape hyrax | ABRQ01717982 | 748-1863 | - | AY374520 | 9e-25 | 7.9e-32 | 477-1127 | EBLN-6 (NP) |
| (*Procavia capensis*) | ABRQ01025914 | 899-1961 | - | FJ169441 | 4e-10 | 6.9e-07 | 417-959 |  |
|  | ABRQ01490479 | 23-517 | + | DQ680833 | 8e-24 | 1.5e-30 | 633-1127 |  |
|  | ABRQ01638802 | 2207-2653 | - | AY374520 | 2e-22 | 2e-28 | 681-1127 |  |
|  | ABRQ01487990 | 1859-2518 | - | AY374533 | 2e-06 | 5e-07 | 90-803 |  |
|  | ABRQ01625290 | 61-314 | - | FJ620690 | 2e-10 | 5.1e-14 | 492-743 |  |
| Mouse | NC_000072 | 87894067-87895116 | + | AY374519 | 2e-58 | 1.3e-61 | 66-1160 |  |
| (*Mus musculus*) | NC_000077 | 103490049-103490965 | - | BDU94863 | 7e-57 | 2.4e-62 | 201-1151 |  |
|  | NC_000077 | 93851147-93851916 | + | X68392 | 1e-33 | 6e-34 | 351-1154 |  |
|  | NC_000084 | 67195203-67196049 | + | FJ792853 | 2e-45 | 2.4e-49 | 201-1037 |  |
|  | NC_000085 | 30608578-30610889 | + | EU781967 | 4e-107 | 4.5e-19 | 4707-6998 | EBLL-7 (L) |
| Rat | NC_005109 | 82624202-82624951 | + | AF136236 | 2e-53 | 1e-53 | 135-1052 |  |
| (*Rattus norvegicus*) | NC_005109 | 92784697-92785239 | - | AY374519 | 6e-70 | 4.3e-66 | 174-1181 |  |
|  | NC_005117 | 63553447-63554378 | + | AY374519 | 6e-70 | 1.2e-58 | 135-1154 |  |
|  | NC_005103 | 122161250-122161697 | + | BDU94875 | 5e-24 | 6.9e-25 | 672-1019 |  |
|  | NC_005100 | 57747112-57747381 | + | FJ169440 | 6e-19 | 1.4e-25 | 606-875 |  |
|  | NC_005100 | 234382241-234384470 | + | EU781967 | 1e-99 | 4.5e-13 | 4860-7217 | EBLL-7 (L) |
| Ground squirrel  (*Spermophilus tridecemlineatus*) | AAQQ01780169 | 444-1538 | + | EU781967 | 2e-160 | 2.5e-188 | 171-1157 |  |
| Guinea pig | AAKN02042182 | 3227-3797 | + | AY374542 | 7e-26 | 3.7e-37 | 435-1061 |  |
| (*Cavia porcellus*) | AAKN02025587 | 41988-42592 | + | AY374542 | 0.038 | 4.4e-08 | 534-881 |  |
| Ord’s kangaroo rat  (*Dipodomys ordii*) | ABRO01003680 | 2720-3221 | - | AY374542 | 1e-07 | 3.9e-10 | 534-1013 |  |
| Bat [19] | AAPE01532006 | 713-1725 | + | FJ002319 | 5e-15 | 9e-19 | 534-971 |  |
| (*Myotis lucifugus*) | AAPE01270722 | 11354-12283 | + | FJ620690 | 2e-14 | 7.2e-18 | 534-1022 |  |
|  | AAPE01525870 | 60-975 | + | FJ002319 | 4e-13 | 1.4e-14 | 534-971 |  |
|  | AAPE01357976 | 3569-5199 | - | FJ169440 | 8e-104 | 2.3e-24 | 4551-6182 |  |
|  | AAPE01357976 | 659-2240 | - | AY114163 | 1e-91 | 2.5e-23 | 4560-6182 |  |
|  | AAPE01358378 | 103-1227 | + | FJ169440 | 1e-103 | 1.3e-23 | 5220-6182 |  |
| Tenrec  (*Echinops telfairi*) | AAIY01076406 | 5259-6303 | - | - | - | - | 642-953 |  |
| Common shrew | AALT01408031 | 4690-5190 | - | FJ792853 | 2e-13 | 5e-18 | 534-1022 |  |
| (*Sorex araneus*) | AALT01464110 | 430-888 | + | EU781967 | 5e-11 | 3.5e-14 | 534-971 |  |
| Opossum | AAFR03032811 | 17552-18109 | - | FJ169441 | 6e-15 | 1.7e-13 | 483-848 |  |
| (*Monodelphis domestica*) | AAFR03030551 | 158351-161130 | + | FJ620690 | 1e-93 | 6.2e-50 | 5448-7625 |  |
|  |  |  |  |  |  |  |  |  |

**Table S3.** Endogenous viral elements related to negative sense RNA viruses (continued)

| Host species ^1^ | Contig ^2^ | Location ^3^ | 4 | Best viral match ^5^ | NR  e-value ^6^ | PFAM  e-value ^7^ | Genomic  region ^8^ | Element name ^9^ |
| --- | --- | --- | --- | --- | --- | --- | --- | --- |
| ***Filoviridae*** |  |  |  |  |  |  |  |  |
| *Ebolavirus* |  |  |  |  |  |  | Ebola Reston |  |
| Little brown bat | AAPE01395781 | 2282-3337 | - | EU338380 | 1e-44 | 1.5e-65 | 578-1612 |  |
| (*Myotis lucifugus*) | AAPE01196249 | 5481-6192 | - | FJ217161 | 1e-35 | 2.9e-52 | 908-1558 |  |
|  | AAPE01378617 | 1-791 | + | EU224440 | 4e-29 | 8.4e-44 | 635-1420 |  |
|  | AAPE01395344 | 1071-1859 | + | FJ621585 | 2e-31 | 2.2e-38 | 3384-4148 |  |
|  | AAPE01428956 | 2563-2823 | - | FJ743676 | 2e-15 | 1.8e-21 | 1190-1450 |  |
| Tammar wallaby | ABQO010277157 | 693-1503 | - | FJ621584 | 1e-43 | 4.7e-71 | 680-1498 |  |
| (*Macropus eugenii*) | ABQO010766573 | 360-1038 | + | FJ217162 | 2e-44 | 2.1e-67 | 938-1624 |  |
|  | ABQO010381849 | 1091-1858 | - | AY729654 | 2e-43 | 2.4e-60 | 875-1645 |  |
|  | ABQO010804673 | 293-1000 | - | FJ750955 | 1e-56 | 1.6e-85 | 911-1624 |  |
|  | ABQO010048551 | 2831-3489 | + | FJ217161 | 4e-30 | 1.2e-46 | 635-1231 |  |
|  | ABQO010309592 | 8756-9234 | - | AY058895 | 9e-33 | 1.2e-46 | 914-1411 |  |
|  | ABQO010478145 | 77-684 | + | FJ968794 | 8e-21 | 2.4e-29 | 911-1621 |  |
|  | ABQO010853818 | 816-1789 | - | FJ621583 | 2e-36 | 9.4e-64 | 647-1630 |  |
|  | ABQO010223025 | 1997-2783 | - | FJ621583 | 1e-16 | 3.7e-24 | 659-1636 |  |
|  | ABQO010212046 | 2720-3151 | + | FJ217162 | 2e-22 | 3.7e-32 | 911-1396 |  |
|  | ABQO010378232 | 12-561 | - | FJ621585 | 7e-09 | 5.2e-14 | 3852-4642 |  |
|  | ABQO011152526 | 11-220 | + | DQ447660 | 5e-14 | 2.7e-25 | 1343-1552 |  |
|  | ABQO010047515 | 2130-2437 | - | FJ743676 | 6e-14 | 1.3e-23 | 1214-1531 |  |
| Opossum | AAFR03010417 | 72682-75762 | - | AY769362 | 1e-79 | 9.5e-15 | 13384-15570 |  |
| (*Monodelphis domestica*) | NW_001581868 | 13311127-13311826 | + | AY769362 | 4e-41 | 9.3e-68 | 992-1696 |  |
| Tarsier | ABRT010013008 | 11185-11704 | - | FJ217161 | 7e-15 | 7.8e-16 | 3621-4148 |  |
| (*Tarsius syrichta*) |  |  |  |  |  |  |  |  |
| Kangaroo rat  (*Dipodomys ordii*) | ABRO01205910 | 1645-2013 | + | FJ217161 | 7e-13 | 1.5e-19 | 1205-1558 |  |
| Guinea pig  (*Cavia porcellus*) | AAKN02025587 | 32716-33015 | + | GQ499199 | 3e-10 | 6.5e-17 | 1193-1462 |  |
| Common shrew  (*Sorex araneus*) | AALT01167855 | 1786-2199 | - | FJ217161 | 1e-12 | 4.3e-09 | 923-1447 |  |
|  |  |  |  |  |  |  |  |  |
| ***Rhabdoviridae*** |  |  |  |  |  |  |  |  |
| *Vesiculovirus* |  |  |  |  |  |  | Vesicular stomatitis virus |  |
| Blacklegged tick | ABJB010759113 | 1728-7719 | - | AY074803 | 0.0 | 7.8e-238 | 4832-10690 |  |
| (*Ixodes scapularis*) | ABJB010703762 | 2-3664 | - | AY074804 | 0.0 | 6.9e-99 | 6827-10417 |  |
|  | ABJB010465412 | 3-1226 | + | EU373658 | 8e-91 | 4.9e-33 | 7523-8695 |  |
|  | ABJB010142368 | 141-1115 | + | AY074804 | 4e-71 | 7.3e-44 | 5582-6553 |  |
|  | ABJB010532089 | 4-2151 | - | EU373657 | 7e-177 | 2.2e-140 | 4832-6913 |  |
|  | ABJB010430317 | 553-1791 | - | EF612701 | 1e-52 | 1.9e-77 | 142-1305 |  |
|  | ABJB010725326 | 3239-4489 | - | EF612701 | 3e-52 | 4.5e-88 | 118-1302 |  |
|  | ABJB010759113 | 8220-9458 | - | EF612701 | 1e-55 | 8.1e-74 | 142-1305 |  |
|  | ABJB010440982 | 3146-4372 | - | EF612701 | 1e-42 | 1.1e-75 | 118-1305 |  |
|  | ABJB010059001 | 41-724 | - | AY840978 | 1e-35 | 2.5e-52 | 634-1305 |  |
|  | ABJB010430317 | 553-1791 | - | EF612701 | 1e-52 | 1.9e-77 | 142-1305 |  |
| Yellow fever mosquito | AAGE02003866 | 28955-29876 | + | EF173366 | 8e-158 | 3.3e-94 | 6053-6928 |  |
| *(Aedes aegypti)* | AAGE02000295 | 45909-46376 | + | FJ665628 | 4e-30 | 7.1e-37 | 8259-8729 |  |
|  | AAGE02024228 | 4748-5132 | + | EU373657 | 2e-37 | 5.2e-22 | 5876-6262 |  |
|  | AAGE02005401 | 11447-11842 | - | AJ810084 | 2e-25 | N/A | 9617-10003 |  |
|  | AAGE02028516 | 19322-21042 | + | AJ810084 | 1e-114 | 1.2e-46 | 7259-8998 |  |
|  | AAGE02000109 | 10351-11601 | + | AF523194 | 8e-45 | 7e-66 | 142-1107 |  |
|  | AAGE02016722 | 26179-27345 | - | AF523194 | 6e-39 | 1.3e-66 | 241-1254 |  |
|  | AAGE02001215 | 25835-26779 | - | AF523194 | 1e-40 | 1.1e-65 | 277-1107 |  |
|  | AAGE02023708 | 4461-5684 | + | AF523194 | 9e-40 | 2.7e-61 | 277-1107 |  |
|  | AAGE02002666 | 109714-110964 | - | U10363 | 1e-41 | 2.8e-66 | 277-1137 |  |
|  | AGE02013358 | 304758-305708 | + | DQ227502 | 5e-27 | 1.8e-45 | 3285-4244 |  |
|  | AAGE02013358 | 306294-307052 | + | DQ227502 | 3e-24 | 1.8e-43 | 3285-4064 |  |
|  | AAGE02013358 | 328857-329471 | + | FJ872827 | 7e-22 | 3.5e-39 | 3285-3929 |  |
|  | AAGE02013358 | 327843-328457 | + | U18101.2 | 7e-22 | 1.5e-39 | 3285-3929 |  |
|  | AAGE02011785 | 82630-83397 | - | U18101.2 | 6e-24 | 5.9e-41 | 3285-4073 |  |
|  | AAGE02018553 | 29534-30604 | - | FJ872827 | 2e-28 | 2e-46 | 3285-4364 |  |
|  | AAGE02018553 | 46857-47561 | - | AY614723 | 5e-10 | 3.9e-19 | 3090-3833 |  |

**Table S3.** Endogenous viral elements related to negative sense RNA viruses (continued)

| Host species ^1^ | Contig ^2^ | Location ^3^ | 4 | Best viral match ^5^ | NR  e-value ^6^ | PFAM  e-value ^7^ | Genomic  region ^8^ | Element name ^9^ |
| --- | --- | --- | --- | --- | --- | --- | --- | --- |
| ***Rhabdoviridae*** |  |  |  |  |  |  |  |  |
| *Vesiculovirus* |  |  |  |  |  |  | Vesicular stomatitis virus |  |
|  | AGE02029253 | 1631-2845 | + | D26175 | 6e-25 | 6.9e-38 | 3129-4340 |  |
|  | AGE02029253 | 49623-50666 | + | FJ985749 | 4e-20 | 1.3e-39 | 3237-4274 |  |
|  | AGE02029253 | 12615-13760 | + | AY614720 | 1e-22 | 2.9e-35 | 3129-4274 |  |
|  | AAGE02001422 | 25867-26910 | - | FJ872827 | 8e-21 | 2.7e-42 | 3237-4274 |  |
|  | AAGE02016690 | 36382-36918 | + | FJ872827 | 6e-23 | 4.1e-39 | 3285-3851 |  |
|  | AGE02000021 | 44030-44884 | - | FJ872827 | 1e-17 | 1.2e-33 | 3237-4094 |  |
|  | AAGE02017532 | 41701-42467 | + | EU370915 | 2e-23 | 2e-33 | 3285-4073 |  |
|  | AAGE02010785 | 64243-65127 | + | AY614723 | 5e-16 | 3.8e-31 | 3093-3998 |  |
|  | AAGE02010562 | 116044-116697 | + | FJ872827 | 1e-12 | 6.3e-26 | 3387-3851 |  |
|  | AAGE02010564 | 9823-10526 | + | D26175 | 1e-15 | 5.7e-31 | 3285-3851 |  |
|  | AAGE02000022 | 2897-3247 | + | AM690337.2 | 3e-05 | 1e-10 | 3732-4073 |  |
| Southern house mosquito | AAWU01016427 | 47658-48827 | + | AF523194 | 5e-24 | 8.4e-47 | 223-1314 |  |
| (*Culex quinquefasciatus*) |  |  |  |  |  |  |  |  |
|  |  |  |  |  |  |  |  |  |
| ***Orthomyxoviridae*** |  |  |  |  |  |  |  |  |
| *Quarjavirus* |  |  |  |  |  |  | Quaranfil virus hemagglutinin gene |  |
| Blacklegged tick | ABJB010794785 | 7898-9823 | - | FJ861694 | 6e-80 | 8.1e-24 | 145-1416 |  |
| (*Ixodes scapularis*) |  |  |  |  |  |  |  |  |
|  |  |  |  |  |  |  |  |  |
| ***Bunyavirus*** |  |  |  |  |  |  |  |  |
| *Nairovirus* |  |  |  |  |  |  | Crimean-Congo hemorrhagic fever segment S |  |
| Blacklegged tick | ABJB010497283 | 5-1402 | + | M86624 | 2e-18 | 2.5e-09 | 167-1501 |  |
| (*Ixodes scapularis*) | ABJB011017495 | 309-881 | - | AF504294 | 6e-18 | 8.5e-09 | 923-1501 |  |
|  | ABJB010284780 | 10845-11495 | - | AF504294 | 7e-15 | 1.4e-11 | 965-1501 |  |
|  | ABJB010495030 | 1303-1845 | - | AF504294 | 3e-16 | 1.2e-09 | 953-1501 |  |
|  | ABJB010498712 | 800-1372 | - | AF504294 | 3e-15 | 1.7e-10 | 923-1501 |  |
|  |  |  |  |  |  |  |  |  |
| *Phlebovirus* |  |  |  |  |  |  | Uukuniemi segment S |  |
| Blacklegged tick [7] | ABJB010470763 | 553-1110 | - |  |  |  | 50-604 |  |
| (*Ixodes scapularis*) | ABJB010497108 | 118-681 | - | M33551 | 1e-60 | 3.2e-21 | 47-604 |  |
|  | ABJB010547597 | 5011-5487 | - | EF201817 | 4e-11 | 8.7e-18 | 50-520 |  |
|  | ABJB010116450 | 644-1204 | - | EF201818 | 2e-09 | 4.8e-20 | 47-601 |  |
|  | ABJB010284780 | 8704-9246 | - | EF201818 | 43-05 | 1.3e-10 | 185-520 |  |
|  |  |  |  |  |  |  | Uukuniemi segment L |  |
| Blacklegged tick | ABJB010460423 | 8633-9634 | - | D10759 | 3e-29 | 3.9e-08 | 1208-2248 |  |
| (*Ixodes scapularis*) | ABJB010108447 | 4353-5566 | - | D10759 | 3e-58 | 3.8e-13 | 1589-2656 |  |
|  |  |  |  |  |  |  |  |  |

**Table footnote:**

^1^ Common name of host species. Numbers in parentheses indicate the total number of matches identified where only a subset are shown. ^2^ GenBank accession number of the contig containing the EVE sequence. ^3^ Location of EVE sequence within contig. ^4^EVE orientation relative to contig. ^5^ Accession number and ^6^ e-value of best matching of best matching viral sequence, based on tBLASTn search against Genbank with putative EVE peptides (see methods section). ^7^ e-value of putative EVE peptide sequence to top-scoring PFAM database viral match (*^a^* removed stop codons). ^8^ Location of EVE nucleotide sequence relative to type species virus of the most closely related virus genus, based on pairwise tBLASTn with EVE peptide. ^9^ Element names are shown for elements that were orthologous across one or more host taxa (see methods section). Names follow the convention of Horie *et al* for Bornavirus-related elements).
