# Supplementary material for: Endogenous Viral Elements in Animal Genomes
Source: PLoS Genet. 2010 Nov 18;6(11):e1001191. doi: 10.1371/journal.pgen.1001191 (PMC2987831; doi:10.1371/journal.pgen.1001191)
Supplement: Table S4 — Endogenous viral elements related to doubled-stranded RNA viruses. (0.03 MB DOC) [file pgen.1001191.s007.doc]

**Table S4.** Endogenous viral elements related to doubled-stranded RNA viruses

| Host species ^1^ | Contig ^2^ | Location ^3^ | 4 | Best viral match ^5^ | NR  e-value ^6^ | PFAM  e-value ^7^ | Genomic  region ^8^ |
| --- | --- | --- | --- | --- | --- | --- | --- |
| ***Reoviridae*** |  |  |  |  |  |  |  |
| *Seadornavirus* |  |  |  |  |  |  | **Liaoning virus segment 5** |
| Yellow fever mosquito | AAGE02000021 | 8314-9021 | -ve | AY317103.1 | 2e-86 | 4e-14 | 241-1746 |
| *(Aedes aegypti)* |  |  |  |  |  |  |  |
|  |  |  |  |  |  |  |  |

**Table footnote:**  See footnote for table S3.
