# Supplementary material for: Endogenous Viral Elements in Animal Genomes
Source: PLoS Genet. 2010 Nov 18;6(11):e1001191. doi: 10.1371/journal.pgen.1001191 (PMC2987831; doi:10.1371/journal.pgen.1001191)
Supplement: Table S5 — Endogenous viral elements related to positive sense RNA viruses. (0.09 MB DOC) [file pgen.1001191.s008.doc]

**Table S5.** Endogenous viral elements related to positive sense RNA viruses

| Host species ^1^ | Contig ^2^ | Location ^3^ | 4 | Best viral match ^5^ | NR  e-value ^6^ | PFAM  e-value ^7^ | Genomic  region ^8^ |
| --- | --- | --- | --- | --- | --- | --- | --- |
|  |  |  |  |  |  |  |  |
| *Flaviviridae* |  |  |  |  |  |  |  |
| *Flavivirus* |  |  |  |  |  |  | **Kamiti River virus** |
| Yellow fever mosquito | AAGE02013358 | 350998-356394 | +ve | AY149905 | 0.0 | 5.1e-41 | 2683-8113 |
| *(Aedes aegypti)* |  | 362100-373970 | +ve | AY149905 | 1e-123 | 2e-35 | 2422-5988 |
|  |  | 373911-375703 | +ve | AY149905 | 7e-143 | 2.1e-42 | 4525-5988 |
|  |  | 375653-376273 | +ve | AY149905 | 4e-06 | - | 3643-3936 |
|  |  | 379637-380470 | +ve | AY149905 | 7e-78 | 3e-21 | 5191-5988 |
|  |  | 380411-385681 | +ve | AY149905 | 9e-20 | - | 3409-5988 |
|  |  | 385622-387353 | +ve | AY149905 | 9e-54 | 2.3e-10 | 3463-4887 |
|  |  | 394622-396299 | +ve | AY149905 | 3e-73 | 1.7e-15 | 2536-3936 |
|  |  | 417592-417780 | +ve | AY149905 | 1e-21 | 9.1e-11 | 7627-7815 |
|  | AAGE02011785 | 74913-78549 | -ve | AY149905 | 3e-105 | 1.3e-58 | 154-3753 |
|  | AAGE02010562 | 52134-53624 | +ve | AY149905 | 5e-154 | 8.8e-168 | 8161-9537 |
|  |  | 84484-84930 | +ve | AY149905 | 4e-53 | 1.5e-66 | 9457-9912 |
|  | AAGE02020997 | 3-632 | -ve | AY149905 | 2e-68 | 1.1e-61 | 2683-3303 |
|  |  | 741-1019 | -ve | AY149905 | 2e-21 | 2.1e-15 | 2680-2922 |
|  |  | 1128-1406 | -ve | AY149905 | 5e-21 | 1.1e-14 | 2680-2922 |
|  |  | 1566-2639 | -ve | AY149905 | 4e-64 | 8e-44 | 2680-3699 |
|  |  | 2799-3632 | -ve | AY149905 | 4e-56 | 8.6e-43 | 2938-3699 |
|  |  | 3679-3867 | -ve | AY149905 | 6e-14 | 4.6e-6 | 2689-2868 |
|  |  | 4027-5927 | -ve | AY149905 | 4e-86 | 1.5e-61 | 2683-3699 |
|  |  | 5206-5484 | -ve | AY149905 | 4e-21 | 3e-15 | 2680-2922 |
|  |  | 5644-6714 | -ve | AY149905 | 1e-84 | 4.9e-62 | 2683-3699 |
|  |  | 6823-7101 | -ve | AY149905 | 4e-21 | 3e-15 | 2680-2922 |
|  |  | 7261-8286 | -ve | AY149905 | 8e-75 | 3e-59 | 2683-3699 |
|  |  | 8386-8673 | -ve | AY149905 | 9e-21 | 7.1e-15 | 2689-2922 |
|  |  | 8782-9060 | -ve | AY149905 | 5e-21 | 1.1e-14 | 2680-2922 |
|  | AAGE02020998 | 1-735 | -ve | AY149905 | 2e-60 | 1.2e-59 | 2683-3396 |
|  |  | 768-835 | -ve | AY149905 | 7e-21 | 1.9e-15 | 2689-2922 |
|  |  | 1282-2352 | -ve | AY149905 | 6e-85 | 1.7e-61 | 2683-3699 |
|  |  |  |  |  |  |  |  |

**Table footnote:**  See footnote for table S3.
