# Supplementary material for: Endogenous Viral Elements in Animal Genomes
Source: PLoS Genet. 2010 Nov 18;6(11):e1001191. doi: 10.1371/journal.pgen.1001191 (PMC2987831; doi:10.1371/journal.pgen.1001191)
Supplement: Table S6 — Endogenous viral elements related to single stranded DNA viruses. (0.21 MB DOC) [file pgen.1001191.s009.doc]

**Table S6. Endogenous viral elements related to single stranded DNA viruses**

| Host species ^1^ | Contig ^2^ | Location ^3^ | 4 | Best viral match ^5^ | NR  e-value ^6^ | PFAM  e-value ^7^ | Genomic  region ^8^ | Element name ^9^ |
| --- | --- | --- | --- | --- | --- | --- | --- | --- |
| ***Parvoviridae*** |  |  |  |  |  |  |  |  |
| ***Genus Dependovirus*** |  |  |  |  |  |  | **AAV2** |  |
| Domestic dog | NC_006619 | 12272147-12272509 | - | DQ335246 | 3e-36 | 4.5e-33 | 4045-4356 |  |
| (*Canis familiaris*) | NC_006621 | 74798635-74798781 | - | EU583391 | 2e-05 | 1.1e-08 | 1323-1469 |  |
| Guinea pig (8) | AAKN02035362 | 8370-9796 | + | DQ335246.2 | 4e-168 | 1.6e-87 | 321-1760 |  |
| (*Cavia porcellus*) | AAKN02031205 | 114399-115225 | + | DQ335246.2 | 2e-43 | 3.5e-26 | 330-1208 |  |
|  | AAKN02030352 | 3872-5256  11742-12062 | + | AY742934 | 2e-42 | 3.1e-22 | 969-2637 |  |
|  | AAKN02045644 | 16301-19700 | - | DQ335246 | 2e-22 | 2.7e-12 | 934-4338 |  |
|  | AAKN02032906 | 58198-58707 | - | DQ196319 | 5e-33 | 2.1e-19 | 1206-1721 |  |
| Nine-banded armadillo | AAGV020719236 | 1855-2469 | - | AY242998 | 4e-74 | 3.4e-56 | 2950-3681 |  |
| (*Dasypus novemcinctus*) |  |  |  |  |  |  |  |  |
| Horse | NC 009151 | 1277165-1277545 | - | EF515837 | 5e-09 | 8.1e-12 | 1236-1475 |  |
| (*Equus caballus*) | NC 009175 | 77091065-77091265 | - | AF416726 | 2e-12 | 4.8e-31 | 1275-1670 |  |
| Tammar wallaby (11) | ABQO010585939 | 126-4049 | + | AY388617 | 0.0 | 5.9e-123 | 330-4386 |  |
| (*Macropus eugenii*) | ABQO010091390 | 1491-2329 | - | U48704 | 2e-61 | 1.8e-25 | 3604-4410 |  |
|  | ABQO010903052 | 518-1113 | + | FJ688147 | 3e-56 | 1.8e-46 | 3037-3642 |  |
|  | ABQO010889914 | 572-1923 | + | GQ368252 | 8e-74 | 1.9e-31 | 510-1826 |  |
|  | ABQO010481652 | 712-1284 | + | AY530611 | 7e-40 | 1.1e-17 | 3682-4242 |  |
|  | ABQO010585938 | 1-333 | + | GQ368252 | 3e-17 | 2.7e-22 | 336-668 |  |
|  | ABQO010444976 | 2723-3869 | - | AY390557 | 4e-62 | 7.9e-20 | 1410-2673 |  |
|  | ABQO010059570 | 4449-5075 | - | U22967 | 3e-25 | 3.4e-09 | 783-1532 |  |
|  | ABQO011172433 | 48-525 | - | X75093 | 3e-23 | 4.1e-06 *^a^* | 702-1202 |  |
|  | ABQO010958468 | 613-795 | + | AY695375 | 1e-13 | 7.6e-12 *^a^* | 1323-1505 |  |
| African elephant  (*Loxodonta Africana*) | AAGU03013549 | 51509-53236 | + | DQ335246 | 0.0 | 1.3e-112 | 330-1841 |  |
| Mouse | NC_000069 | 12016997-12020624 | - | DQ335246 | 9e-68 | 6.5e-20 | 1026-4410 |  |
| (*Mus musculus*) | NC_000074 | 95686602-95687837 | - | AF416726 | 2e-09 | 9.2e-07 | 1317-2613 |  |
|  | NC_000067 | 194639536-194639781 | + | J01902 | 2e-06 | 0.004 | 618-881 | EVE-DV1 |
| Little brown bat | AAPE01526173 | 3215-682 | - | AY631965 | 0.0 | 6.9e-83 | 318-4410 |  |
| (*Myotis lucifugus*) | AAPE01230204 | 1592-1783 | + | AY530577 | 1e-35 | 5.8e-18 | 3637-4410 |  |
|  | AAPE01230202 | 518-1284 | - | AY530606 | 4e-13 | 6.3e-08 | 4219-4410 |  |
|  | AAPE01291520 | 6586-6927 | - | DQ335246 | 2e-09 | 1.4e-11 | 1314-1625 |  |
| Pika | AAYZ01294085 | 5975-6766 | - | AF085716 | 1e-16 | 2.5e-11 | 780-1472 |  |
| (*Ochotona princeps*) |  |  |  |  |  |  |  |  |
| Duckbilled platypus | AAPN01125634 | 7183-7479 | - | DQ250134 | 7e-12 | 1.7e-14 | 1413-1715 |  |
| (*Ornithorhynchus* | AAPN01022475 | 2333-2680 | + | EF515837 | 4e-09 | 4.3e-05 | 1233-1583 |  |
| *Anatinus*) | AAPN01206586 | 909-1194 | + | AY530625 | 2e-06 | 2.6e-10 | 3046-3324 |  |
|  | AAPN01206585 | 357-390 | + | AY388617 | 4e-04 | 0.022 | 1389-1490 |  |
| European rabbit  *Oryctolagus cuniculus* | AAGW02036031 | 4287-7892 | + | FJ688147 | 1e-122 | 1.1e-53 | 354-4374 |  |
| Hamadyras baboon  (*Papio hamadryas*) | Contig290628-  Contig638931 | 117545-119924 | - | AY695376 | 0.0 | 1.9e-107 | 339-2721 |  |
|  | Contig185865 | 216-738 | + | U48704 | 2e-67 | 0.053 | 1854-2376 |  |
|  | Contig190611-Contig189280 | 9000-10344 | + | AY695374 | 0.0 | 9.1e-99 | 321-1688 |  |
| Cape hyrax | ABRQ01260357 | 188-970 | - | AY388617 | 4e-69 | 1.5e-28 | 396-1253 |  |
| (*Procavia capensis*) | ABRQ01135041 | 4588-4770 | - | AY530574 | 2e-16 | 2.3e-07 | 4207-4389 |  |
|  | ABRQ01135041 | 4754-4966 | - | AY530616 | 1e-10 | 0.0019 | 4030-4221 |  |
|  | ABRQ01135041 | 4827-5198 | - | AY530595 | 6e-19 | 0.0026 | 3790-4149 |  |
|  | ABRQ01135041 | 5579-5848 | - | AY530575 | 9e-06 | 0.0026 | 4045-4284 |  |
|  | ABRQ01135041 | 5998-6327 | + | AY243026 | 2e-24 | 4.8e-14 | 2587-2982 |  |
| Malayan flying fox | ABRP01003662 | 2591-2824 | - | AY629582 | 6e-07 | 6.5e-11 | 1296-1532 |  |
| (*Pteropus vampyrus*) | ABRP01170809 | 859-1059 | - | AY629583 | 8e-07 | 5.1e-09 | 1287-1463 |  |
|  | ABRP01157241 | 13665-13959 | - | DQ269987 | 7e-25 | 7.1e-11 | 981-1304 |  |
| Brown rat | NC_005112.2 | 108702300-108702830 | + | AF513851 | 1e-23 | 5.3e-07 | 330-845 | EVE-DV1 |
| (*Rattus norvegicus*) | NC_005101.2 | 91480723-91481022 | + | AF028704 | 8e-15 | 1.1e-05 *^a^* | 1011-1328 |  |
|  | NC_005118.2 | 14969560-14969913 | + | AY388617 | 1e-07 | 0.28*^a^* | 1374-1727 |  |
|  | NC_005104.2 | 65632931-65633263 | + | X01457.1 | 2e-43 | 3.2e-31 | 2332-2646 |  |
| Bottlenose dolphin | ABRN01283281 | 1468- 3175 | + | EU253479 | 9e-108 | 3.6e-68 | 354-4374 |  |
| (*Tursiops truncatus*) | ABRN01191161 | 9009-9371 | - | GQ200736 | 2e-07 | 4.9e-09 | 1311-1436 |  |
| Alpaca (*Vicugna pacos*) | ABRR01368792 | 4082-4485 | + | AY530593 | 8e-32 | 3.8e-14 | 3997-4398 |  |

**Table S6. Endogenous viral elements related to single stranded DNA viruses (continued)**

| Host species ^1^ | Contig ^2^ | Location ^3^ | 4 | Best viral match ^5^ | NR  e-value ^6^ | PFAM  e-value ^7^ | Genomic  region ^8^ | Element name ^9^ |
| --- | --- | --- | --- | --- | --- | --- | --- | --- |
|  |  |  |  |  |  |  |  |  |
| ***Parvoviridae*** |  |  |  |  |  |  |  |  |
| ***Genus Parvovirus*** |  |  |  |  |  |  | **MVM** |  |
| Guinea pig (5)  (*Cavia porcellus*) | AAKN02030352 | 3872-5256  11213-13835 | + | AY742934 | 8e-169 | 3.4e-55 | 288-4452 |  |
|  | AAKN02055888 | 79584-82768 | + | AY390557 | 3e-64 | 4.7e-23 | 1200-4413 |  |
|  | AAKN02032906 | 58083-59816 | - | U34253 | 3e-63 | 5.7e-23 | 297-1862 |  |
|  | AAKN02032908 | 10674-12353 | + | AF036710 | 9e-58 | 1.4e-25 | 306-1862 |  |
| Tenrec  (*Echinops telfairi*) | AAIY01487966 | 1828-2527 | - | AF036710 | 9e-45 | 1.1e-11 | 1131-1838 |  |
| Rat | NC_005104.2 | 65636489-65635512 | - | AF036710 | 2e-114 | 5.4e-38 | 261-1103 |  |
| (*Rattus norvegicus*) |  | 65632586-65635106 | + |  |  | 5.1e-143 | 2100-4557 |  |
| Tammar wallaby (28) | ABQO010318785 | 1-1818 | - | FJ822038 | 8e-79 | 3e-60 | 1278-3036 |  |
| (*Macropus eugenii*) | ABQO010519946 | 60-2355 | + | AB437434 | 9e-84 | 7.6e-70 | 2431-4527 |  |
|  | ABQO010334457 | 1750-4391 | + | AY684869 | 5e-85 | 4.5e-68 | 1719-4428 |  |
|  | ABQO010193462 | 47-1429 | - | AY390557 | 3e-54 | 6.3e-64 | 3055-4428 |  |
|  | ABQO010065506 | 1048-2591 | - | EU498687 | 2e-57 | 1.2e-50 | 2923-4440 |  |
| Opossum (6) | NC_008803 | 352563141-352567160 | - | FJ592174 | 8e-58 | 8.8e-42 | 279-4431 |  |
| (*Monodelphis domestica*) | NC_008806 | 48166623-48171573 | + | AY684870 | 9e-96 | 5.1e-70 | 6-4425 |  |
|  | NC_008808 | 230386981-230396815 | + | AY390557 | 2e-78 | 7.2e-46 | 645-4431 |  |
|  | NC_008806 | 113564918-352567160 | + | U34256 | 5e-63 | 5.1e-39 | 1338-2646 |  |
|  |  |  |  |  |  |  |  |  |
| *Genus Amdovirus* |  |  |  |  |  |  | **AMDV** |  |
| Cape hyrax | ABRQ01360977 | 3625-3945 | + | X97629 | 4e-13 | 3e-19 | 2538-2855 |  |
| (*Procavia capensis*) |  |  |  |  |  |  |  |  |
|  |  |  |  |  |  |  |  |  |
| ***Circoviridae*** |  |  |  |  |  |  |  |  |
| ***Genus Circovirus*** |  |  |  |  |  |  | **PCV-1** |  |
| Domestic dog | **NW_876275** | 5737517-5738450 | + | AJ298230 | 7e-16 | 0.00048 *^a^* | 92-832 |  |
| *(Canis familiaris)* | **NW_876263** | 34420784-34420897 | + | AF311299 | 7e-07 | 0.0011 *^a^* | 647-760 |  |
|  | **NW_876313** | 83572-84058 | - | DQ915950 | 2e-19 | 1.2e-07 *^a^* | 371-847 | EVE-CV1 |
| Cat | **ACBE01536005** | 794-1486 | + | AF311299 | 3e-11 | 0.0003 *^a^* | 275-826 |  |
| *(Felis cattus)* | **ACBE01511791** | 1129-1325 | + | DQ915960 | 8e-10 | No match | 644-832 | EVE-CV1 |
| Giant panda  (*Ailuropoda melanoleuca*) | **scaffold 9548*** | 91-741 | + | GQ404844 | 7e-28 | 7.5e-10 *^a^* | 281-919 | EVE-CV1 |
| Opossum | **NW_001581902** | 9462550-9463357 | - | FJ623185 | 2e-49 | 3e-17 *^a^* | 89-982 |  |
| *(Monodelphis domestica)* |  |  |  |  |  |  |  |  |

**Table footnote:**  See footnote for table S3. Abbreviations: AAV=adeno-associated virus; MVM=minute virus virus; AMDV=Aleutian mink disease virus; PCV-1=porcine circovirus type-1.
