# Supplementary material for: Endogenous Viral Elements in Animal Genomes
Source: PLoS Genet. 2010 Nov 18;6(11):e1001191. doi: 10.1371/journal.pgen.1001191 (PMC2987831; doi:10.1371/journal.pgen.1001191)
Supplement: Table S7 — Endogenous viral elements related to reverse transcribing DNA viruses. (0.04 MB DOC) [file pgen.1001191.s010.doc]

**Table S7.** Endogenous viral elements related to reverse transcribing DNA viruses

| Host species ^1^ | Contig ^2^ | Location ^3^ | 4 | Best viral match ^5^ | NR  e-value ^6^ | PFAM  e-value ^7^ | Genomic  region ^8^ |
| --- | --- | --- | --- | --- | --- | --- | --- |
| *Hepadnavirus* |  |  |  |  |  |  | Duck hepatitis B virus |
| Zebrafinch  *(Taeniopygia guttata)* | chromosome 1  (NW_002197691) | 7791931-7792098 | + | AJ251937 | 2e-05 | 2.8e-05 | 85-249 |
|  |  | 7792885-7793886 | + | AF110996 | 3e-18 | 0.025 | 2198-2521 |
|  | chromosome 12  (NW_002197249) | 1842426-1842938 | + | AY494852 | 2e-35 | 8e-38 | 269-787 |
|  | chromosome 26  (NW_002198080) | 864217-864447 | - | AF110997 | 2e-17 | 1.5e-10 | 440-667 |
|  | chromosome Z  (NW_002234454) | 2740404-2742565 | + | AY433937 | 1e-140 | 1.1e-27 | 1313-2524 |
|  | chromosome 20  (NW_002197828) | 1111835-1112362 | - | AY494849 | 2e-46 | 2.8e-06 | 1391-1864 |
|  |  |  |  |  |  |  |  |
|  | chromosome 24  (NW_002198024) | 1452555-1452825 | + | M22056 | 3e-14 | 0.015 | 2791-3027 |

**Table footnote:**  See footnote for table S3.
